# Pulmonary inflammation decreases with ultra-protective ventilation in experimental ARDS under VV-ECMO: a positron emission tomography study

**DOI:** 10.3389/fmed.2024.1338602

**Published:** 2024-02-20

**Authors:** Guillaume Deniel, François Dhelft, Sophie Lancelot, Maciej Orkisz, Emmanuel Roux, William Mouton, Nazim Benzerdjeb, Jean-Christophe Richard, Laurent Bitker

**Affiliations:** ^1^Service de Médecine Intensive-Réanimation, Hôpital de la Croix-Rousse, Hospices Civils de Lyon, Lyon, France; ^2^Univ Lyon, Université Claude Bernard Lyon 1, INSA-Lyon, CNRS, Inserm, CREATIS UMR, Villeurbanne, France; ^3^Université de Lyon, Université LYON 1, Lyon, France; ^4^CERMEP – Imagerie du Vivant, Lyon, France; ^5^Hospices Civils de Lyon, Lyon, France; ^6^Laboratoire Commun de Recherche Hospices Civils de Lyon/bioMérieux, Centre Hospitalier Lyon Sud, Hospices Civils de Lyon, Lyon, France; ^7^Centre d’Anatomie et Cytologie Pathologique, Centre Hospitalier Lyon Sud, Hospices Civils de Lyon, Lyon, France

**Keywords:** acute respiratory distress syndrome, [^11^C](R)-PK11195, ventilator-induced lung injury, positron emission tomography, ultra-protective ventilation, quantitative computerized tomography

## Abstract

**Background:**

Experimentally, ultra-protective ventilation (UPV, tidal volumes [V_T_] < 4 mL.kg^−1^) strategies in conjunction with veno-venous extracorporeal membrane oxygenation (VV-ECMO) are associated with lesser ventilator-induced lung injuries (VILI) during acute respiratory distress syndrome (ARDS). However, whether these strategies reduce lung inflammation more effectively than protective ventilation (PV) remains unclear. We aimed to demonstrate that a UPV strategy decreases acute lung inflammation in comparison with PV in an experimental swine model of ARDS.

**Methods:**

ARDS was induced by tracheal instillation of chlorhydric acid in sedated and paralyzed animals under mechanical ventilation. Animals were randomized to receive either UPV (V_T_ 1 mL.kg^−1^, positive end-expiration pressure [PEEP] set to obtain plateau pressure between 20 and 25 cmH_2_O and respiratory rate [RR] at 5 min^−1^ under VV-ECMO) or PV (V_T_ 6 mL.kg^−1^, PEEP set to obtain plateau pressure between 28 and 30 cmH_2_O and RR at 25 min^−1^) during 4 h. After 4 h, a positron emission tomography with [^11^C](R)-PK11195 (ligand to TSPO-bearing macrophages) injection was realized, coupled with quantitative computerized tomography (CT). Pharmacokinetic multicompartment models were used to quantify regional [^11^C](R)-PK11195 lung uptake. [^11^C](R)-PK11195 lung uptake and CT-derived respiratory variables were studied regionally across eight lung regions distributed along the antero-posterior axis.

**Results:**

Five pigs were randomized to each study group. Arterial O_2_ partial pressure to inspired O_2_ fraction were not significantly different between study groups after experimental ARDS induction (75 [68–80] mmHg in a PV group vs. 87 [69–133] mmHg in a UPV group, *p* = 0.20). Compared to PV animals, UPV animals exhibited a significant decrease in the regional non-aerated compartment in the posterior lung levels, in mechanical power, and in regional dynamic strain and no statistical difference in tidal hyperinflation after 4 h. UPV animals had a significantly lower [^11^C](R)-PK11195 uptake, compared to PV animals (non-displaceable binding potential 0.35 [IQR, 0.20–0.59] in UPV animals and 1.01 [IQR, 0.75–1.59] in PV animals, *p* = 0.01). Regional [^11^C](R)-PK11195 uptake was independently associated with the interaction of regional tidal hyperinflation and regional lung compliance.

**Conclusion:**

In an experimental model of ARDS, 4 h of UPV strategy significantly decreased lung inflammation, in relation to the control of V_T_-derived determinants of VILI.

## 1 Introduction

Low tidal volume (V_T_) ventilation decreases the mortality rate in acute respiratory distress (ARDS) (1). This strategy aims to reduce ventilator-induced lung injuries (VILI), mainly by decreasing lung stress and strain (2). However, under protective ventilation (PV), pneumothorax (significant acute lung injury indicator) is still documented in 10–15% of patients with ARDS (3, 4). Although dynamic strain remains low under protective ventilation, tidal hyperinflation probably causes lung injuries even at V_T_ 6 mL.kg^−1^ in ARDS patients (5, 6).

Moreover, biotrauma (i.e., pulmonary and systemic inflammation induced by mechanical ventilation) may lead to multi-organ failure and has been documented under protective ventilation (7, 8). Ultra-protective ventilation (UPV) strategy (i.e., V_T_ decrease <4 mL.kg^−1^) with veno-venous extracorporeal membrane ventilation (VV-ECMO) was shown to reduce mechanical power (i.e., mecanotrauma) and histologic lung injuries (i.e., VILI) in animals with experimental ARDS (9). However, the impact of these ventilation strategies on mecanotrauma on the one hand and lung (and systemic) biotrauma on the other hand remains poorly described (10–13).

Positron emission tomography (PET) with [^11^C](R)-PK11195 (ligand to TSPO-bearing macrophages) coupled with quantitative computerized tomography (CT) allows the non-invasive and specific quantification of lung macrophages, in parallel with the *in vivo* assessment of CT-derived parameters quantifying VILI, such as tidal hyperinflation and lung strain (11, 14, 15). As macrophage recruitment is a key mechanism in VILI, TSPO ligands are particularly relevant compared with [^18^F] fluoro-2-deoxy-D-glucose (as lung uptake is not specific to a particular cell population) (16).

We hypothesized that UPV would decrease acute lung macrophagic inflammation in swine with experimental ARDS, in relation to a decrease in V_T_-related biomechanical injury, compared to conventional protective ventilation. The main objective of the study was to evaluate the effect on acute lung macrophagic inflammation of a UPV strategy aiming to increase PEEP and decrease V_T_, respiratory rate, and plateau pressure in experimental ARDS, assessed *in vivo* by the regional lung uptake of [^11^C](R)-PK11195, compared to conventional protective ventilation.

## 2 Materials and methods

### 2.1 Animal conditioning

**Figure 1:**
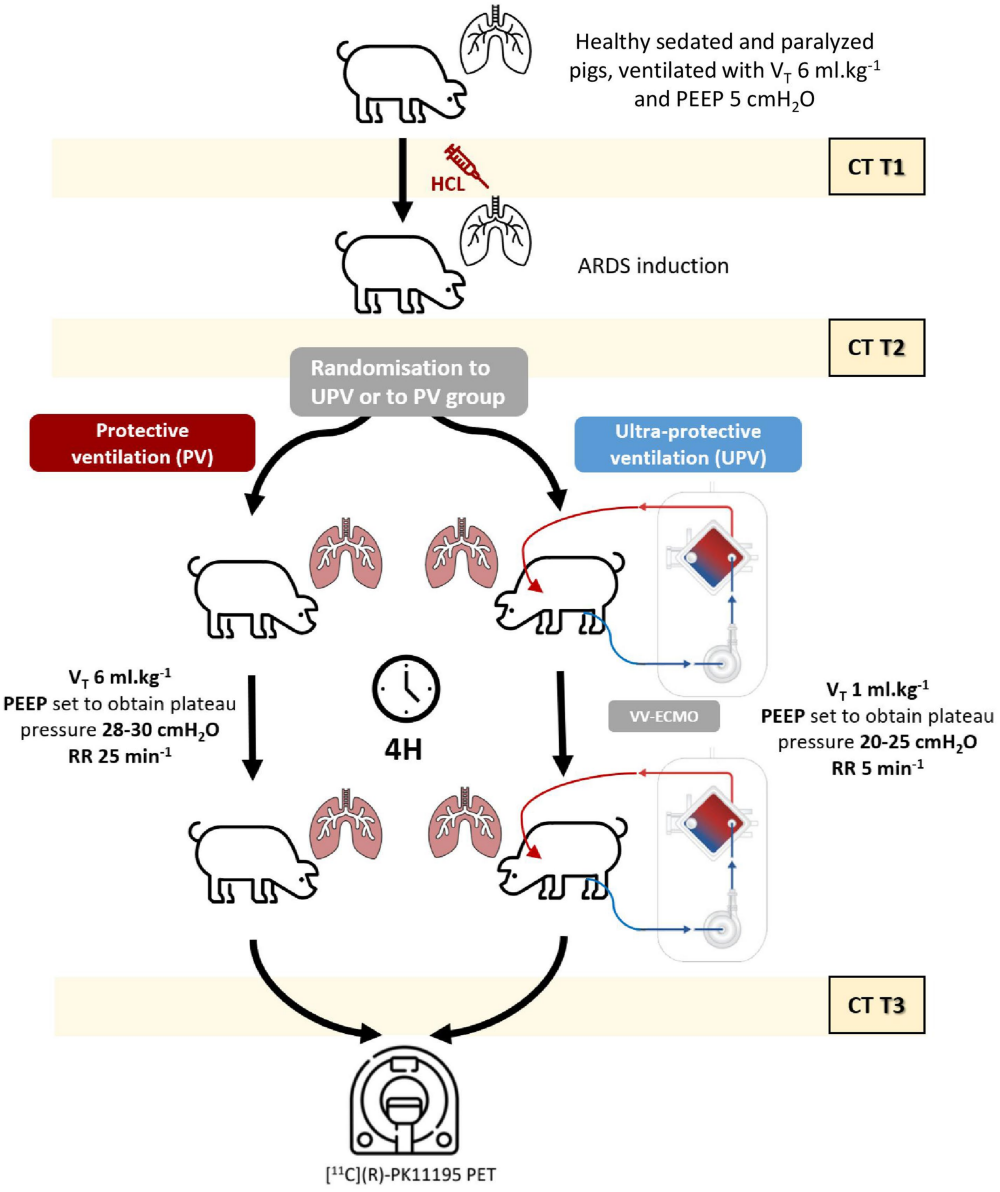
Study protocol. The figure details the protocol process with the three main study steps (before experimental ARDS [T1], after experimental ARDS stabilization [T2], and 4 h later [T3]). CT study designates the 3 CT acquisitions performed at end-expiration, end-inspiration, and FRC in all animals. The PET study designates the [^11^C](R)-PK11195 acquisition performed after 4 h of ventilator settings according to the study group (UPV and control group). ARDS designates the ARDS onset (performed between T1 and T2). Following T3, the animals were euthanized. ARDS, acute respiratory distress syndrome; BW, body weight; CT, computerized tomography; FiO_2_, inspired fraction in O_2_; FRC, functional residual capacity; EI, end-inspiratory airway plateau pressure; PEEP, positive end-expiratory pressure; PET, positron emission tomography; PV, protective ventilation; RR, respiratory rate; UPV, ultra-protective ventilation strategy; V_T_, tidal volume.

The protocol time points and principal measurements are presented in Figure 1. Briefly, after surgical conditioning under general anesthesia, all pigs involved in the study received conventional protective ventilation using the following settings: V_T_ 6 mL.kg^−1^ of body weight (BW), an external positive end-expiratory pressure (PEEP) of 5 cmH_2_O, a respiratory rate of 25 breaths.min^−1^, an inspiratory:expiratory ratio of 1:2, constant inspiratory flow, and an inspired fraction (FiO_2_) of 0.21 O_2_ (1). Animals were then randomized using a pre-determined random list to receive a UPV strategy with either VV-ECMO (UPV group) or conventional protective ventilation (PV group) for 4 h. In the UPV group, VV-ECMO cannulation was performed before experimental ARDS induction to apply the intervention ventilatory strategy immediately after ARDS induction, as in the control group. Circuits were primed with 0.9% saline prior to connection and with heparin (bolus 100 UI.kg^−1^ followed by a continuous perfusion of 10 UI.kg^−1^.h^−1^) at the time of connection. The pump flow was set to deliver a flow of 65 mL.kg^−1^.min^−1^ (17). The sweep gas flow was initially set to 0 mL.min^−1^ until the end of ARDS induction.

Experimental ARDS was induced by the intra-tracheal instillations of a maximum of 5 mL.kg^−1^ of 0.1 M chlorhydric acid. Experimental ARDS was confirmed once the PaO_2_/FiO_2_ ratio was <150 mmHg 30 min after induction. Complete details regarding swine conditioning, VV-ECMO settings, and ARDS induction procedures are presented in the Supplementary material.

### 2.2 Experimental strategies

Animals randomized to the PV group received the following ventilation strategies (without VV-ECMO): V_T_ 6 mL.kg^−1^ of body weight (BW), positive end-expiration pressure (PEEP) set to maintain the end-inspiratory airway plateau pressure between 28 and 30 cmH_2_O (18), a respiratory rate (RR) 25 breaths.min^−1^, an inspiratory:expiratory ratio of 1:2, a constant inspiratory flow, and an inspired fraction in O_2_ (FiO_2_) of 1 (1, 19).

In the UPV group, the sweep gas flow was set to equal VV-ECMO blood flow and adjusted to maintain PaCO_2_ between 35 and 45 mmHg (17), and the O_2_ membrane fraction (FdO_2_) was set to 1. V_T_ was then decreased down to 1 mL.kg^−1^ BW by steps of 1 mL.kg^−1^ BW while progressively increasing PEEP to maintain the end-inspiratory airway plateau pressure between 20 and 25 cmH_2_O, over a period of 30 min. RR was lowered to 5 breaths.min^−1^ once a V_T_ of 1 mL.kg^−1^ BW was reached, with an inspiratory:expiratory ratio of 1:2, a constant inspiratory flow, and FiO_2_ of 1. In both groups, mechanical ventilation settings remained unchanged over the 4 h following randomization, until PET-CT acquisitions. Complete details regarding experimental strategies are presented in the Supplementary material.

### 2.3 CT imaging protocol

CT scans were acquired using a BioGraph TruePoint^®^ PET/CT camera (Siemens, Munich, Germany) before experimental ARDS (T1), after experimental ARDS stabilization (T2), and 4 h later (T3). At each study point, three CT scans were realized: at end-expiration, at end-inspiration, and after transient disconnection from the ventilator (i.e., at functional residual capacity). End-expiration and end-inspiration CT acquisitions were performed after hermetically clamping the tracheal tube (using 2 Kocher clamps).

Lung regions of interest (ROIs) were drawn on all CT scans, excluding major airways, bullae, and pneumothoraxes (20), using CreaTools software (21). To perform regional CT analysis, lung ROIs were divided into 10 regions of equal thickness along the antero-posterior axis. The most anterior and the most posterior ones were excluded to avoid small-volume ROIs and partial volume effect resulting from the vicinity with the chest wall. Regional analyses were therefore performed on the 8 remaining lung levels. The following variables were determined and normalized to the animal body weight (BW, in kg): lung tissue weight at end-expiration, end-expiratory lung volume (EELV), CT-derived V_T_ (V_T,CT_), PEEP-related gas volume (V_PEEP_), tidal lung recruitment and PEEP-related lung recruitment, tidal and end-expiratory hyperinflation, dynamic and static strain, and regional respiratory system compliance at end-expiration (C_RS,PEEP_). Complete details regarding CT imaging protocol and quantitative lung analysis are presented in the Supplementary material.

### 2.4 Quantification of [^11^C](R)-PK11195 lung uptake and study primary outcome

A 60-min PET acquisition with [^11^C](R)-PK11195 was performed after applying the ventilation strategy for 4 h in each animal. At the beginning of the acquisition, the animal received a 5 MBq.kg^−1^ BW [^11^C](R)-PK11195 bolus via the central venous catheter.

[^11^C](R)-PK11195 lung uptake was quantified using a two-tissue compartment (2TCM) or a three-tissue compartment kinetic model (3TCM), as previously described (14). In the 3TCM, a third compartment is considered, in which the tracer is non-specifically and irreversibly fixed, with subsequent significant improvement in the specific quantification of [^11^C](R)-PK1119 (22). This model requires the determination of the following variables: the fraction of whole blood volume in the ROI (F_WB_), parent tracer’s net influx from the plasma pool to tissue expressed as *K_1_* (i.e., entry rate constant from blood to tissue) to *k_2_* (backward rate constant of transfer from tissue to blood) ratio, the rate constant representing [^11^C](R)-PK11195 binding to the TSPO receptor (*k_3_* in min^−1^), the rate constant corresponding to its dissociation from its receptor (*k_4_* in min^−1^), and the rate constant of irreversible uptake by the non-specific compartment (*k_5_* in min^−1^). The study’s primary outcome was the regional non-displaceable binding potential of [^11^C](R)-PK11195 (BP_ND_), determined from those models. BP_ND_ is defined as the ratio of specifically bound [^11^C](R)-PK11195 to its non-displaceable tissue concentration at equilibrium, i.e., the ratio of the *k_3_* rate constant to the *k_4_* rate constant (14, 23), which reflects the number of TSPO receptors in the ROI and is independent of the ROI’s tissue gas fraction (14, 24). Full details regarding the CT and PET imaging protocol are presented in the Supplementary material.

### 2.5 Nanostring mRNA multiplex assay and pathology analysis

At the end of the protocol, the animals were euthanized. The lungs were extracted and sampled for the nanostring mRNA multiplex assay and pathology analysis. The following mRNA were quantified: TSPO (i.e., the protein target of [^11^C](R)-PK11195), CD68 (protein expressed on the surface of macrophages), FCGR3A/B (mRNA produced by natural killer cells and neutrophils), CD84 (protein expressed on the surface of M1 macrophages), CD163 (protein expressed on the surface of M2 macrophages), IL10 (a cytokine promoting M2 phenotype), and IL6 (cytokine promoting M1 phenotype). The distinction between M1 and M2 phenotypes is determinant. Indeed, M1 macrophages have a pro-inflammatory phenotype, participate in the recruitment of other inflammatory cells (polynuclear cells and lymphocytes), and contribute to lung injuries; however, M2 macrophages have an anti-inflammatory phenotype and limit pro-inflammatory cytokine secretion as well as the recruitment of other inflammatory cells (25).

Concerning the pathology analysis, histologic lung injuries were evaluated on the right lung samples using published semi-quantitative scores by a pathologist (NB) blinded to the intervention (6, 11). Complete details regarding mRNA quantification and pathology analysis are presented in the Supplementary material.

### 2.6 Sample size calculation

Based on a 0.7 decrease (standard deviation: ±0.35) in [^11^C](R)-PK11195 BP_ND_, we calculated that 10 animals would be required to observe a significant difference in BP_ND_ between study groups with an α of 0.05 and a β of 0.20. Due to the high mortality rate of this model (30%), we expected to include 14 animals.

### 2.7 Statistics

All statistical analyses were performed using R software (R foundation for statistical computing, version 4.0.5, Vienna, Austria). The following R packages were used: lme4 (26), mice (27), lmerTest (28), and ggplot2 (29). A *p*-value < 0.05 was considered statistically significant. All quantitative data are reported as a median interquartile range. Whole lung measurements (respiratory mechanics and CT measurements) over time and between groups were compared using mixed effects linear regression, with the animal number as the random effect. The association of lung levels (including a quadratic or an exponential factor if significant) and study group with aeration compartment, CT parameters, and regional BP_ND_ were performed using linear mixed effects regression models, using animal number as a random intercept and lung level as a random slope. Interactions were systematically evaluated. We performed a multivariate analysis of the CT parameters associated with regional BP_ND_ using relevant CT-derived variables: EELV, lung tissue weight at end-expiration, C_RS,PEEP_, static and dynamic strain, end-expiratory hyperinflated lung volume, PEEP-related alveolar recruitment, regional V_T_, tidal hyperinflation, tidal recruitment, and lung region identification. In this analysis, missing CT-derived variables (i.e., tidal hyperinflation) were handled using multiple imputations (with the “predictive mean matching” method in 20 imputed datasets). Complete details regarding statistics are presented in the Supplementary material.

## 3 Results

### 3.1 Experimental ARDS

Fifteen pigs were enrolled and randomized; among them, five in each group reached the end of the protocol and were analyzed. Five animals died before primary end-point analysis because of the severity of lung injuries and/or difficulties in VV-ECMO cannulation (Supplementary Figure S1). Ratios of arterial O_2_ partial pressure (PaO_2_) to FiO_2_, pH, arterial CO_2_ partial pressure, lactates, and hemoglobin were not significantly different between study groups after experimental ARDS induction (Supplementary Table S1). Plateau pressures and lung elastance (El_L_) significantly increased after ARDS onset and were significantly different between study groups (Table 1). Between the period before and after ARDS induction, there was a significant increase in whole lung weight, with a significant difference between study groups. However, EELV significantly decreased, with no difference between the groups (Table 2).

**Table 1:** Respiratory mechanics over time.

	Study group				
Variables	PV *n* = 5	UPV *n* = 5	Effect of group, *p*	Effect of time, *p*	Group × time, *p*
Tidal volume, ml.kg^−1^ BW			–	–	<0.01
T1	6.0 [6.0–6.0]	6.0 [6.0–6.1]			
T2	6.0 [6.0–6.0]	6.0 [6.0–6.1]			
T3	6.0 [6.0–6.0]	1.0 [1.0–1.4]^†,‡^			
Plateau pressure, cmH_2_0			–	–	<0.01
T1	14 [13–14]	13 [12–14]			
T2	24 [24–27]^‡^	20 [18–21]^†,‡^			
T3	28 [28–28]^‡^	23 [22–23]^†,‡^			
Total PEEP, cmH_2_0			–	–	<0.01
T1	6 [6–6]	6 [6–7]			
T2	6 [6–6]	6 [6–7]			
T3	9 [9–10]^‡^	20 [19–20]^†,‡^			
RR, min^−1^			–	–	<0.01
T1	25 [20–25]	20 [20–25]			
T2	25 [25–25]	25 [25–25]			
T3	25 [25–25]	5 [5–9]^†,‡^			
ΔP_AW_, cmH_2_0			–	–	<0.01
T1	8 [7–8]	7 [5–7]			
T2	18 [18–20]^‡^	13 [11–15]^†,‡^			
T3	19 [19–20]^‡^	3 [3–3]^†,‡^			
ΔP_ES,_ cmH_2_0			–	–	<0.01
T1	1 [1–1]	2 [2–2]			
T2	2 [2–3]^‡^	2 [2–3]			
T3	3 [3–3]^‡^	0 [0–0]^†,‡^			
El_RS_			–	–	0.02
T1	41 [37–42]	30 [29–32]			
T2	106 [99–114]^‡^	57 [52–61]^†,‡^			
T3	107 [103–116]^‡^	56 [50–71]^†,‡^			
El_CW_			0.73	0.04	0.17
T1	4 [3–6]	9 [8–10]			
T2	9 [8–17]	12 [7–15]			
T3	17 [16–19]	8 [8–8]			
El_L_			–	–	0.01
T1	33 [31–42]	21 [20–23]			
T2	97 [74–98]^‡^	45 [42–49]^†,‡^			
T3	91 [79–97]^‡^	48 [42–63]^†,‡^			
P_L,El_			<0.01	<0.01	0.15
T1	12 [11–13]	9 [9–10]			
T2	22 [18–23]	15 [14–16]		*	
T3	25 [24–25]	18 [18–20]		*	
Power_RS_, J.min^−1^			–	–	<0.01
T1	5.6 [4.5–5.7]	7.3 [5.6–9.1]			
T2	11.5 [11.4–11.6]^‡^	15.4 [12.1–17.1]^‡^			
T3	11.2 [10.9–11.3]^‡^	0.6 [0.4–1.0]^†,‡^			
Resistance, cmH_2_O.s^−1^			–	–	0.01
T1	3.4 [3–3.5]	4.0 [3.3–4.8]			
T2	10.0 [9.2–11.2]^‡^	16.3 [8.0–20.4]^‡^			
T3	8.2 [6.2–8.7]	2.2 [1.3–3.2]			

Values are median with an interquartile range. Study time points were baseline (T1, normal lungs), after experimental ARDS onset (T2), and 4 h later (T3). Mixed effects linear regressions with study group and study time point as independent variables, and animal identification number as random effect, were used to compare variables at different study times and in the two groups. Interaction of time study with the group was systematically checked for (using bootstrap with 500 simulations). If no interaction was identified, the *p* value of the group effect and the study time effect are given, respectively. In case of significant interaction, a pairwise *post-hoc* multiple comparison was performed to compare groups at each time point on the one side and compare T2 and T3 to T1 in each group, on the other. Multiple comparisons were adjusted for α inflation using the Benjamini and Hochberg method.*p < 0.05 compared with T1 (no interaction with study group); †*p* < 0.05 compared with control group at this study time; ‡*p* < 0.05 compared with T1 in this study group.UPV, ultra-protective ventilation; PV, protective ventilation; ΔP_AW_, difference in airway pressure at end-inspiratory (plateau pressure) and at end-expiratory (total PEEP); ΔP_ES_, difference in esophageal pressure at end-inspiratory and end-expiratory; El_RS_, respiratory system elastance; El_CW_, chest wall elastance; El_L_, lung elastance; P_L,El_, elastance derived plateau transpulmonary pressure; Power_RS_, mechanical power; RR, respiratory rate.

**Table 2:** Computed tomography-derived parameters over time.

	Study group				
Variables	PV *n* = 5	UPV *n* = 5	Effect of group, *p*	Effect of time, *p*	Group × time, *p*
Lung tissue weight at end-expiration, g.kg^−1^ BW			–	–	0.05
T1	16 [15–17]	14 [14–16]			
T2	22 [21–26]^‡^	18 [17–20]^†,‡^			
T3	26 [23–27]^‡^	24 [19–24]^‡^			
End-expiratory aerated lung volume (EELV), ml.kg^−1^ BW			–	–	<0.01
T1	21 [19–23]	27 [23–32]			
T2	15 [15–16]	22 [21–22]			
T3	18 [18–22]	34 [33–56]^†,‡^			
Gas fraction at end-expiration			–	–	0.02
T1	0.58 [0.55–0.58]	0.63 [0.63–0.70]			
T2	0.40 [0.40–0.43]^‡^	0.55 [0.54–0.57]^†,‡^			
T3	0.40 [0.39–0.48]^‡^	0.64 [0.64–0.70]^†^			
End-expiratory hyperinflated volume, ml.kg^−1^ BW			–	–	<0.01
T1	0.2 [0.2–0.2]	0.8 [0.8–1.0]			
T2	0.3 [0.2–0.5]	2.0 [2.0–2.1]			
T3	0.5 [0.4–0.6]	6.0 [4.1–7.4]^†,‡^			
PEEP related increase in gas volume (V_PEEP_), ml.kg^−1^ BW			–	–	<0.01
T1	6.2 [5.3–6.8]	5.6 [5.3–5.6]			
T2	3.4 [3.1–5.1]	3.9 [2.6–4.2]			
T3	5.8 [5.7–8.4]	17.8 [14.6–23.5]^†,‡^			
PEEP-related alveolar recruitment, ml.kg^−1^ BW			–	–	0.02
T1	0.7 [0.5–0.9]	0.3 [0.2–0.6]			
T2	1.2 [0.6–1.6]	0.7 [0.4–0.8]			
T3	0.9 [0.7–1.5]	4.0 [2.5–4.6]^†,‡^			
C_RS_ at PEEP (C_RS,PEEP_), ml.cmH_2_O^−1^.kg^−1^ BW			<0.01	<0.01	0.52
T1	1.0 [1.0–1.2]	1.0 [0.9–1.0]			
T2	0.5 [0.5–0.7]	0.5 [0.2–0.6]		*	
T3	0.9 [0.7–0.9]	1.1 [0.9–1.2]		*	
Tidal volume (V_T,CT_), ml.kg^−1^ BW			–	–	<0.01
T1	5.5 [5.1–5.9]	6.2 [6.2–6.5]			
T2	5.3 [4.9–6.1]	6.3 [6.1–6.8]			
T3	5.4 [4.1–5.8]	1.6 [1.0–2.0]^†,‡^			
Tidal recruitment, ml.kg^−1^ BW			0.04	<0.01	0.22
T1	0.2 [0.2–0.5]	0.1 [0.1–0.3]			
T2	0.8 [0.6–1.0]	0.5 [0.4–0.7]			
T3	1.5 [1.3–1.5]	0.3 [0.2–0.7]		*	
Tidal hyperinflation, ml.kg^−1^ BW			–	–	<0.01
T1	0.1 [0.0–0.1]	0.4 [0.3–0.6]			
T2	0.1 [0.1–0.2]	1.5 [1.3–1.7]^†,‡^			
T3	0.2 [0.1–0.4]	0.6 [0.4–0.6]			
Dynamic strain			–	–	0.01
T1	0.2 [0.2–0.2]	0.2 [0.2–0.3]			
T2	0.3 [0.2–0.3]	0.2 [0.2–0.3]			
T3	0.2 [0.1–0.2]	0.0 [0.0–0.0]^†,‡^			
Static strain			0.16	<0.01	0.06
T1	0.4 [0.3–0.4]	0.3 [0.2–0.3]			
T2	0.3 [0.2–0.3]	0.2 [0.1–0.2]		*	
T3	0.4 [0.4–0.4]	0.5 [0.4–0.5]		*	

Values are median with an interquartile range. Study time points were baseline (T1, healthy lungs), after experimental ARDS onset (T2), and 4 h later (T3). Mixed effects linear regressions with study group and study time point as independent variables, and animal identification number as random effect, were used to compare variables at different study times and in the two groups. Interaction of time study with the group was systematically checked for (using bootstrap with 500 simulations). If no interaction was identified, the *p*-value of the group and the time study are given, respectively. In case of significant interaction, a pairwise *post-hoc* multiple comparison was performed to compare groups at each time point on the one side and compare T2 and T3 to T1 in each group, on the other. Multiple comparisons were adjusted for α inflation using the Benjamini and Hochberg method. In pigs 19 and 21, end-inspiratory CT at T3 were missing, and end-inspiratory measurements were not taken.*p < 0.05 compared to T1 (no interaction with study group); †*p* < 0.05 compared with control group at this study time; ‡*p* < 0.05 compared with T1 in this study group.UPV, ultra-protective ventilation; PV, protective ventilation; C_RS,PEEP_, respiratory system compliance at PEEP; EELV, end-expiratory lung volume; V_PEEP_, PEEP-related increase in gas volume; V_T,CT_, computerized tomography-derived tidal volume; BW, body weight.

### 3.2 VV-ECMO settings

At the end of the protocol in UPV animals, VV-ECMO settings were as follows: blood flow at 69 [63–67] ml.kg^−1^.min^−1^, gas sweep flow of 4.0 [4.0–5.5] L.min^−1^, and FdO_2_ of 1 [1–1].

### 3.3 Effects of UPV on CT parameters

End-inspiratory CT images were unexploitable in 2 UPV animals at the end of the experiment and were, hence, excluded from the analysis. In these two animals, EELV and end-inspiratory lung volumes at this experimental time were equal (i.e., tidal volume was null) due to a probable tracheal tube air leak.

**Figure 2:**
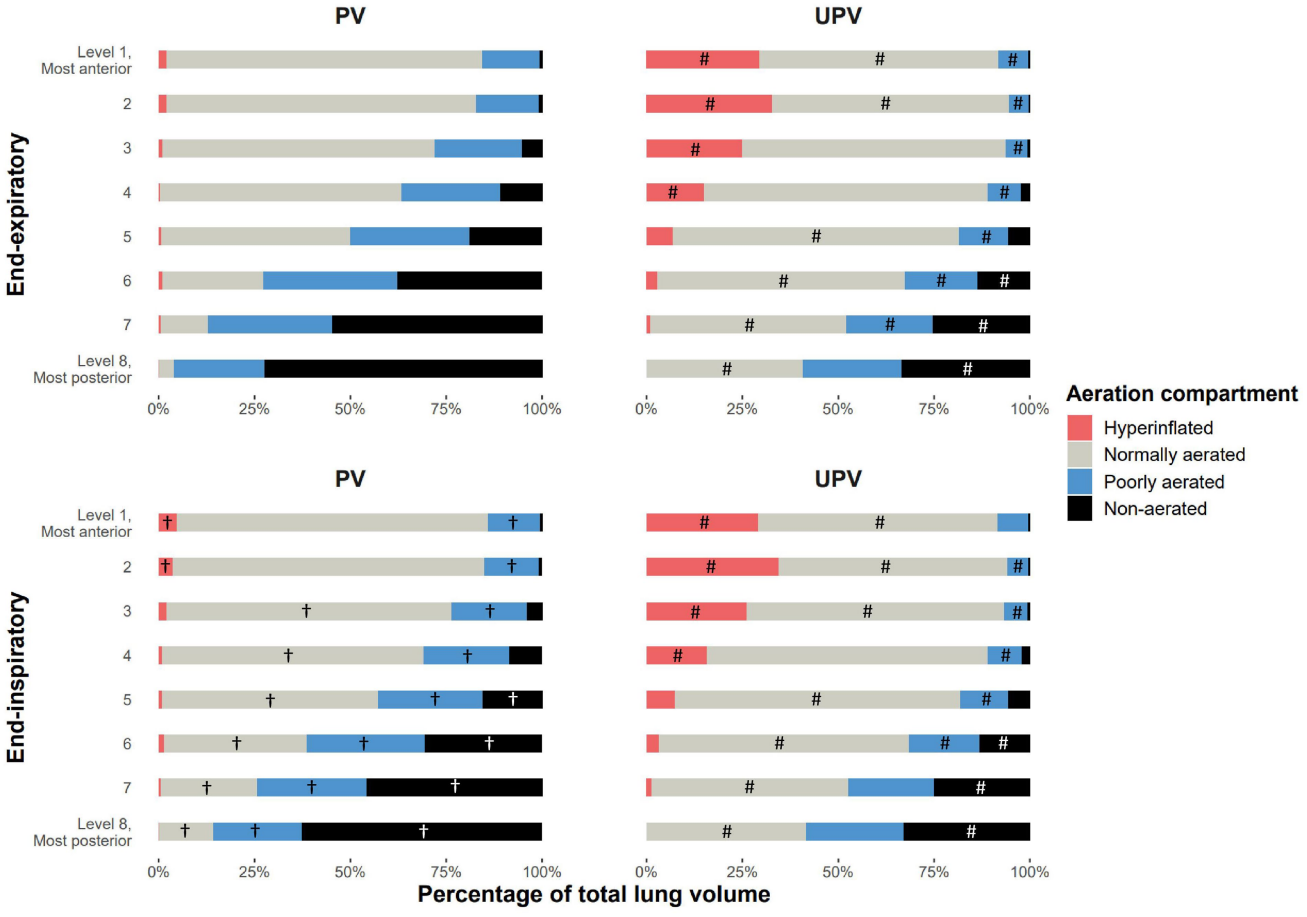
Aeration compartment distribution in lung levels based on the study group at the end of the protocol. The figure shows the mean fraction of each of the four aeration compartments (non-aerated, poorly aerated, normally aerated, and hyperinflated) in each of the eight lung levels of animals in each study group, at end-expiration and end-inspiration, measured after 4 h of the ventilation strategy. The interaction between group and lung levels was significantly associated with the distribution in the total lung volume of the four aeration compartments between the two study groups at end-expiration and between end-expiration and end-inspiration in the PV group. ^#^*p* < 0.05 in *post-hoc* pairwise analysis evaluating the difference in the fraction level for a given aeration compartment in the same lung level, between study groups at end-expiration or end-inspiration (accounting for α risk inflation with the Benjamini and Hochberg method). ^†^*p* < 0.05 in *post-hoc* pairwise analysis evaluating the difference in the fraction level for a given aeration compartment in the same lung level, between respiratory times (end-expiration and end-inspiration) in each study group (accounting for α risk inflation). UPV, ultra-protective ventilation strategy.

**Figure 3:**
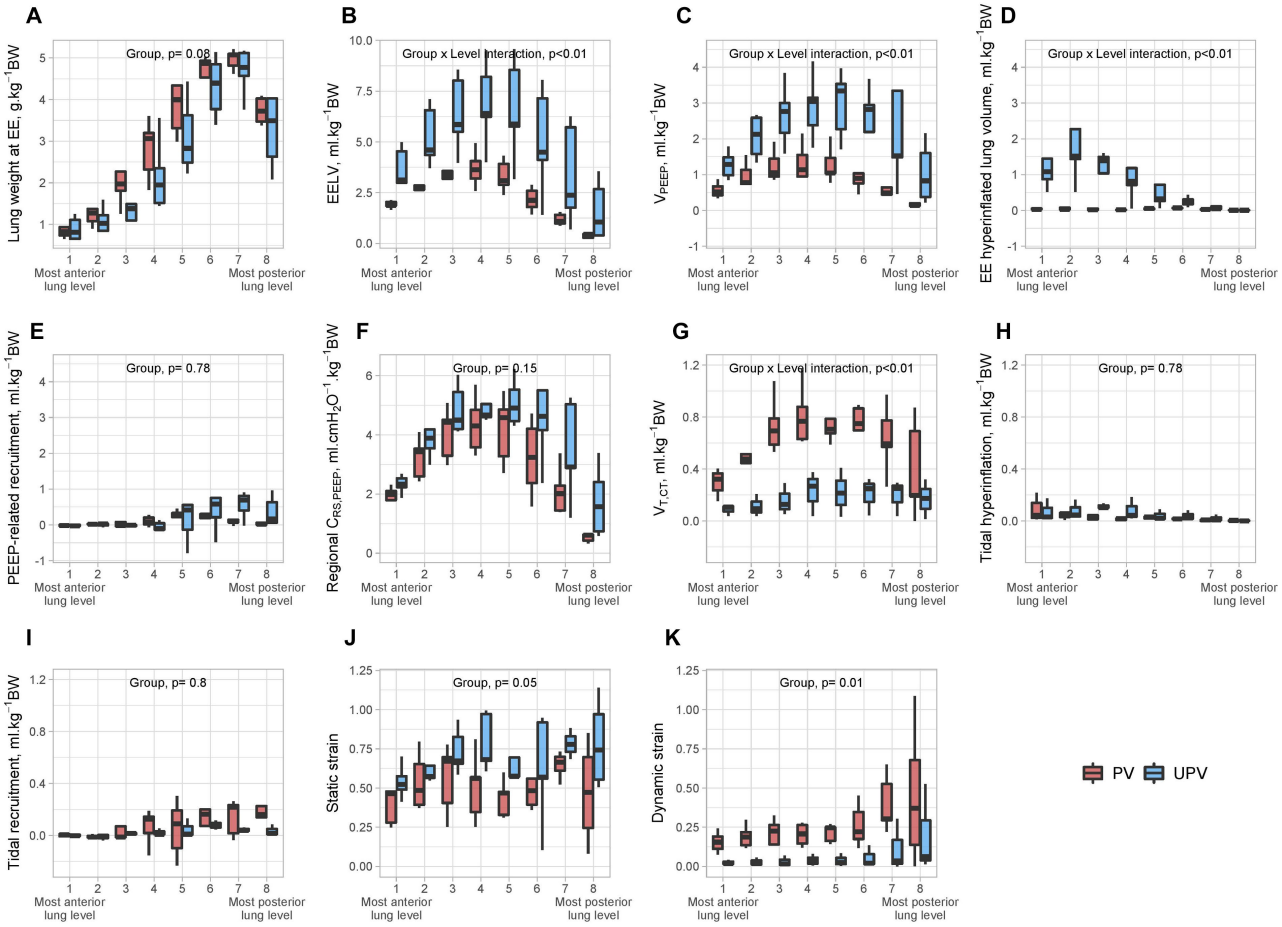
Regional CT parameters in both study groups at the end of the protocol. The figure shows the values of lung weight at end-expiration **(A)**, end-expiratory lung volume **(B)**, V_PEEP_ **(C)**, end-expiratory hyperinflated lung volume **(D)**, PEEP-induced recruitment **(E)**, regional C_RS,PEEP_ **(F)**, V_T,CT_ **(G)**, tidal hyperinflation **(H)**, tidal recruitment **(I)**, static strain **(J)**, and dynamic strain **(K)** in each of eight lung levels, in the UPV group and control. All volumes were normalized to animal body weight. The *p* value examines the association between the studied parameter and interaction of group × lung level (expressed as a second-degree polynomial if statistically significant to account for the non-linearity of the anterior to posterior distribution of some variables). In the case of a non-significant interaction term, the *p* value of the independent effect of the group was given. BW, body weight; C_RS,PEEP_, respiratory system compliance at PEEP; EE, end-expiratory; EELV, end-expiratory lung volume; PEEP, positive end-expiratory pressure; V_T,CT_, computerized tomography-derived tidal volume; V_PEEP_, PEEP-related increase in gas volume; UPV, ultra-protective ventilation strategy.

Animals receiving the UPV strategy had significantly lower V_T,CT_, El_L,_ non-aerated whole lung volume, dynamic strain, and mechanical power (Power_RS_) and significantly higher total PEEP and EELV, compared to PV animals (Tables 1, 2). UPV animals displayed a significant decrease in the regional non-aerated compartment in the posterior lung levels and a significant increase in the hyperinflated compartment in the anterior lung levels compared to PV animals (Figure 2). EELV, V_PEEP_, end-expiratory hyperinflation, regional V_T,CT_, and dynamic strain significantly differed between study groups at the end of the protocol (Figure 3). Absolute tidal hyperinflation was similar in both groups but the fraction of tidal hyperinflation was higher (close to 40% of regional V_T,CT_) in UPV animals (Supplementary Figures S2, S3).

### 3.4 Effect of UPV on [^11^C](R)-PK11195 lung uptake

**Figure 4:**
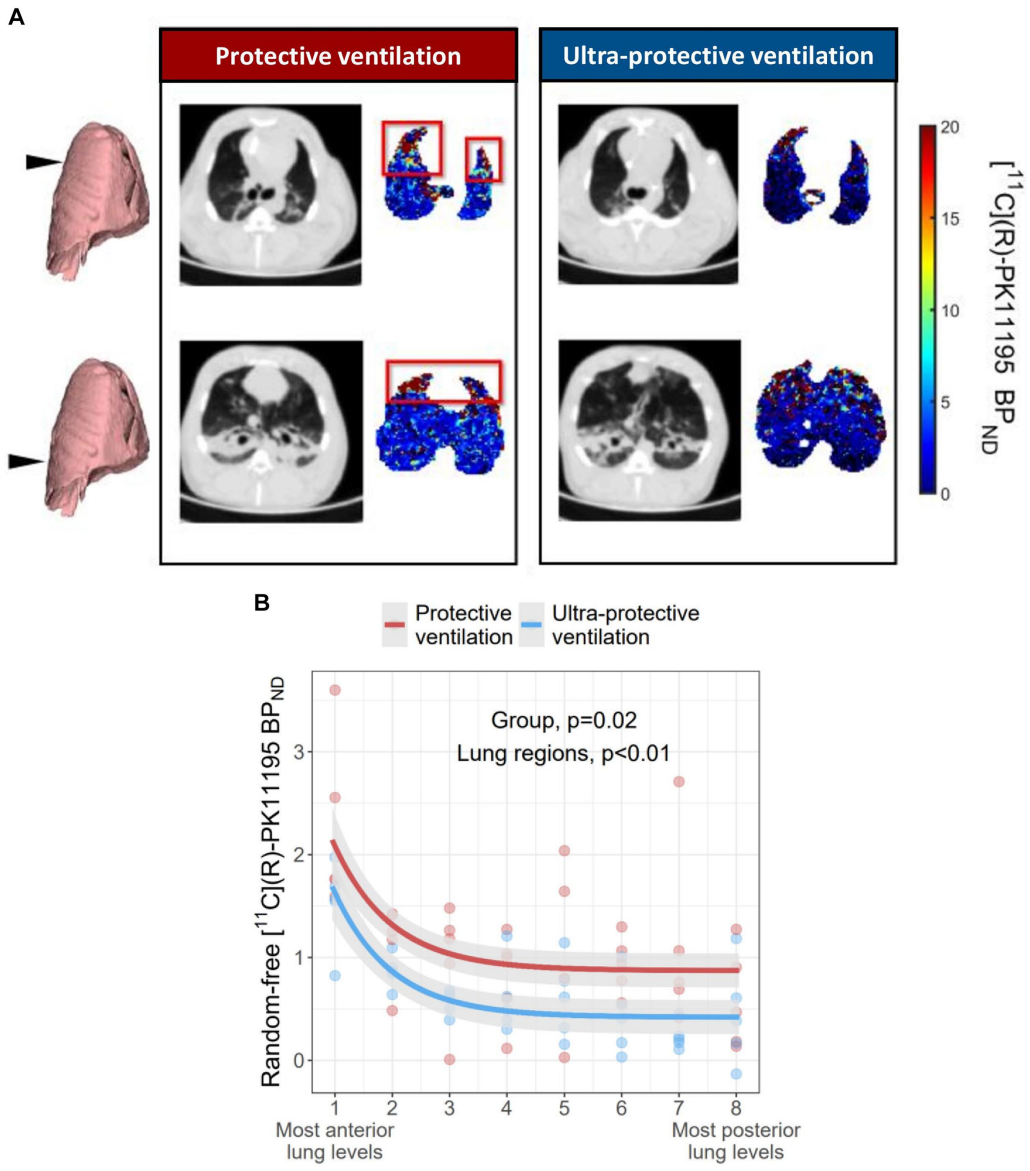
[^11^C](R)-PK11195 BP_ND_ in each lung level in the 2 study groups at the end of the protocol. **(A)** Shows representative [^11^C](R)-PK11195 BP_ND_ parametric images (determined at voxel level using the TCM methodology) of an animal in the UPV group and another of the control group, at two anatomical levels along the cephalocaudal axis of the lung. The color scale describes the voxels [^11^C](R)-PK11195 BP_ND_ values in PET images. Anatomical levels are given with the black arrow on the left and the corresponding end-expiratory CT slice. The red box shows lung regions with high macrophagic inflammation, as estimated by [^11^C](R)-PK11195 BP_ND_. **(B)** Shows an adjusted BP_ND_ of [^11^C](R)-PK11195 in each of the 8 lung levels, in animals of the UPV group and those in the control group. For graphical representation, [^11^C](R)-PK11195 BP_ND_ was adjusted according to the random slopes and intercepts of the lung region and the animal identification number, respectively, determined by the mixed effect regression model. Dots represent standardized experimental data collected at T3 in all animals. The mixed effects model describing the effect of the study group and lung levels on BP_ND_ is represented by the two curves. Shading along the lines represents the 95% confidence interval of the model. The *p* value examines the association between the study group and/or lung regions with regional BP_ND._ Model residuals were checked graphically for normality. The two explanatory variables retained in the final model were the study group and the region (expressed as an exponential). The final model marginal and conditional R^2^ were 0.19 and 0.75, respectively. [^11^C](R)-PK11195 BP_ND_: non-displaceable binding potential; UPV: ultra-protective ventilation strategy.

Whole lung BP_ND_ reached 0.35 [0.20–0.59] in UPV animals and 1.01 [0.75–1.59] in PV animals (*p* = 0.01). Regional BP_ND_ was significantly lower in all lung regions of the UPV group after 4 h than in the PV group (Figure 4). The significant association between the regional BP_ND_ and the study group remained after adjustment for regional lung weight after ARDS induction (*p* = 0.02). Regional BP_ND_ was also significantly higher in anterior lung regions than in posterior ones in both groups (Figure 4). Other 3TCM model parameters at T3 are described in the Supplementary material (Supplementary Results, Supplementary Figure S4).

### 3.5 Determinants of regional lung inflammation

**Figure 5:**
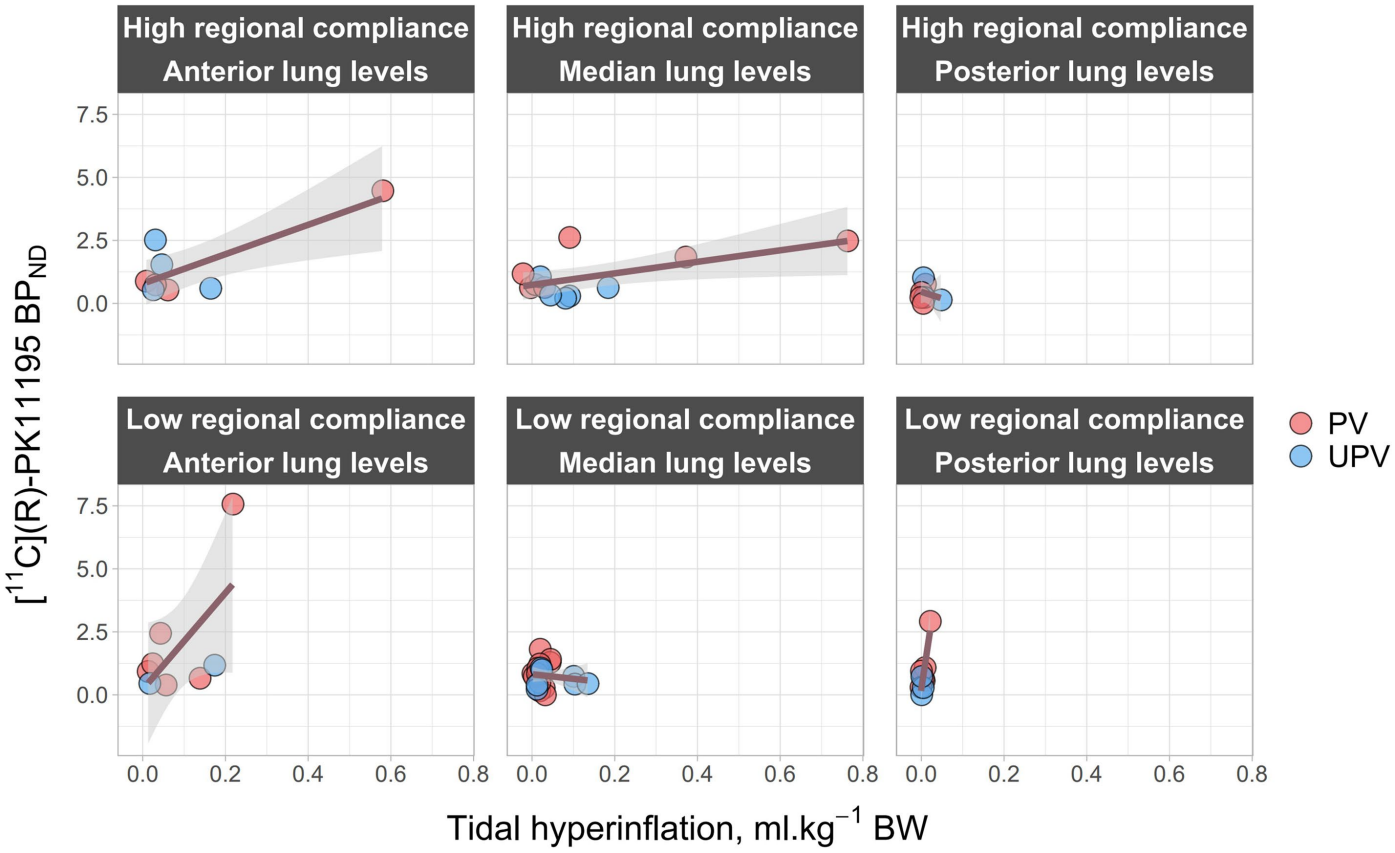
Regional [^11^C](R)-PK11195 BP_ND_ in relation to regional CT parameters at the end of the protocol. The figure represents the effect of the interaction of regional C_RS,PEEP_, lung levels, and tidal hyperinflation with regional, lung inflammation. It shows regional [^11^C](R)-PK11195 BP_ND_ (x-axis) as a function of tidal hyperinflation (y-axis) and across 3 lung levels (“Anterior lung levels” regroups lung levels 1 and 2, “Median lung levels” regroups lung levels 3 to 6 and “Posterior lung levels” regroups lung levels 7 and 8) and 2 regional compliance levels (“Low regional compliance” and “High regional compliance”). Regional compliance levels were defined as “Low” if C_RS,PEEP_ was strictly below its median value in each previously defined lung levels, or as “High” if it was higher than the median value. Dark pink lines represent a linear mixed-effect regression model between tidal hyperinflation and [^11^C](R)-PK11195 BP_ND_ across the 3 lung levels and the 2 regional compliance levels. Gray areas correspond to 95% confidence intervals. PV, protective ventilation; UPV, ultra-protective ventilation; BP_ND_, [^11^C](R)-PK11195 binding potential; BW, body weight; EELV, end-expiratory lung volume; Regional C_RS,PEEP_, regional respiratory system compliance at PEEP; PEEP, positive end-expiratory pressure.

Univariate analysis of BP_ND_ determinants is presented in Supplementary Table S2. In multivariate analysis, regional [^11^C](R)-PK11195 BP_ND_ was significantly and independently associated with the interaction between tidal hyperinflation, C_RS,PEEP_, and lung levels (Figure 5).

### 3.6 Nanostring mRNA multiplex assay and pathology analysis

NanoString^®^ mRNA quantification showed a significant interaction between the study group and the lung region for IL10 expression. There was a non-significant trend in lower TSPO mRNA expression in posterior lung regions of UPV animals compared to PV animals (Supplementary Figure S5). In the pathology study, the acute lung injury score was not different among study groups (Supplementary Figure S6).

### 3.7 Effects of UPV on systemic hemodynamics

At the end of the experiment, UPV animals had significantly higher norepinephrine dose compared with control animals (0.7 [0.3–1.19] mg.h^−1^ vs. 0.0 [0.0–0.0] mg.h^−1^, *p* < 0.01), with no significant difference in cardiac output (4.3 [4.0–5.5] L.min^−1^ vs. 4.2 [3.4–4.6] L.min^−1^, *p* = 0.35). Animals in the UPV groups received more fluids during the protocol (31 [30.3–33.3] ml.kg^−1^ in the UPV group vs. 0 [0–0] ml.kg^−1^ in the PV group, *p* < 0.01). There was a non-significant trend in lower pH and higher lactate levels in the UPV group compared with PV animals (Supplementary Table S1).

## 4 Discussion

In this study, we evaluated the effects of a UPV strategy (with VV-ECMO) combining very low V_T_, high PEEP, low RR, and controlled plateau pressure on regional acute lung inflammation assessed by the lung uptake of [^11^C](R)-PK11195 in an animal model of ARDS. The main findings of the study were as follows: (1) the UPV strategy resulted in a significant reduction in mechanical power, transpulmonary pressures, and improved lung compliance, at the price of a significant increase in hyperinflation in anterior lung regions; (2) this strategy significantly decreased acute macrophagic lung inflammation in all lung regions; and (3) regional acute lung inflammation decreased in relation to the combined effects of regional lung compliance at PEEP decrease and regional tidal hyperinflation increase.

To the best of our knowledge, this is the first study to assess the effect of UPV associating ultra-low V_T_, high PEEP, low RR, and limited plateau pressure on regional lung inflammation evaluated using a validated PET methodology (14). The applied ventilation strategies were in line with the current literature. We designed an intervention group (i.e., UPV group) aiming to decrease all VILI determinants, not just tidal volume. Previous trials aiming to decrease VILI had similar interventions. For example, ARMA’s protocol compared a “traditional ventilation” strategy (i.e., tidal volume at 12 mL.kg^−1^ and plateau pressure under 50 cmH_2_O) to a “protective ventilation” strategy (i.e., tidal volume at 6 mL.kg^−1^ and plateau pressure under 30 cmH_2_O) (1). On the other hand, strategies with only tidal volume decrease (and similar plateau pressure) across the study groups failed to demonstrate survival benefits in ARDS patients under extracorporeal circulation (30).

First, plateau pressures in the UPV group were limited as in EOLIA’s intervention group (i.e., under VV-ECMO) and as those in an experimental model published by Araos et al. (i.e., plateau pressure under 24 cmH_2_O) (9, 31). This lower target aiming to limit lung stress was justified by the documentation of 10 to 15% of pneumothorax (barotrauma surrogate) in ARDS patients receiving protective ventilation (i.e., plateau pressure under 28 cmH_2_O) (4).

The V_T_ decrease in the UPV strategy allowed a significant drop in Power_RS_, total lung stress, and regional dynamic strain values. This aspect is in line with the study of Araos et al., where they compared a near apneic ventilation strategy (V_T_ close to 2 mL.kg^−1^ and RR at 5.min^−1^) to a conventional protective ventilation in an experimental ARDS model of a pig with VV-ECMO (9). They observed similar biomechanical improvement (drop in Power_RS_ and decrease in lung stress) associated with acute lung injury attenuation. Reduction of all known mechanical drivers of VILI is also relevant outside the field of UPV and VV-ECMO, as several studies documented VILI in healthy or ARDS swine models, despite plateau pressure below 25 cmH_2_O, in correlation with Power_RS_ and ventilatory settings (32, 33).

RR reduction (one of the equational determinants of Power_RS_) is known to be associated with lesser lung injuries in ARDS animal models under protective ventilation, but also with mortality in ARDS patients when associated with V_T_ reduction (34). Experimental studies demonstrated the importance of RR in VILI development in ARDS models receiving conventional ventilation strategies (35–37).

However, Power_RS_ may not be the only determinant of VILI development. Indeed, Moraes et al. demonstrated that V_T_ increase with a constant Power_RS_ was associated with an increase in lung injuries (38). We can assume that tidal hyperinflation, a parameter unaccounted for in Power_RS_ computation, could be a determinant of lung injuries. Indeed, Terragni et al. proved that under protective ventilation, some patients with ARDS generated high tidal hyperinflation and that it was associated with a higher biotrauma phenotype (i.e., higher pro-inflammatory cytokine production) (5). We highlighted the association between regional tidal hyperinflation and regional lung inflammation in this study and in several previous studies (6, 11).

Parallel to the beneficial effect of low V_T_, low plateau pressure, and low Power_RS_ on VILI, high PEEP levels generated significant end-expiratory hyperinflation in our model, especially in anterior lung regions, and a significant rise in regional static strains despite non-negligible PEEP-related recruitment. Chlorhydric acid-induced ARDS is known for lower recruitment potential, compared to other highly recruitable models such as oleic acid or saline lavage (39). Regarding the known effects of PEEP on regional lung inflammation, Güldner et al. observed higher regional inflammation in anterior and mid lung regions of animals with high static strain, compared to animals with low static strain, when dynamic strain was kept constant (40). In different ventilation settings, Wellman et al. had similar results to ours regarding lung inflammation (using [^18^F]-FDG), with a “protective” effect of higher regional gas fractions generated by high PEEP associated with low dynamic strain compared with animals exposed to high dynamic strain ventilated at zero PEEP (10). We hypothesize that high PEEP levels and high static strain may have improved regional lung compliance by recruiting atelectatic lung regions to a certain extent and may have subsequently translated into decreased lung inflammation response. Hence, we designed the PV strategy with a PEEP set to maintain plateau pressure between 28 and 30 cmH_2_O, in line with the EXPRESS trial’s protocol (19). On the other hand, higher inflammation in the PV group may be the consequence of targeting this plateau pressure.

Our results unexpectedly showed that high PEEP levels generated large end-expiration lung volumes with end-expiratory hyperinflation and subsequently caused high tidal hyperinflation despite low V_T_ in UPV animals. Indeed, we observed that a high PEEP strategy led to a significantly higher number of voxels with an HU number lying immediately above the definition threshold of −901 HU at end-expiration. These voxels were permuted to the hyperinflated compartment at end-inspiration despite low V_T_, with the subsequent consequence of higher-than-expected relative tidal hyperinflation in this group. Previous experimental studies have shown that tidal hyperinflation was associated with higher lung inflammation and was significantly associated with worse outcomes in critically ill patients ventilated with conventional protective ventilation (5, 10, 11, 41). However, in these studies, tidal hyperinflation was present when V_T_ was equal to or above 6 mL.kg^−1^ BW and at PEEP levels well below those applied in our study. Taken together, our results demonstrate not only the effect of V_T_ on tidal hyperinflation but also its interaction with the resting aerated lung volume determined by PEEP and, more specifically, its hyperinflated component.

Finally, we aimed in this study to combine several validated tools (parametric functional imaging, quantitative CT, mRNA quantification, and pathology) to evaluate lung inflammation. We documented a decrease of IL10 mRNA expression in anterior lung regions, in the UPV group compared to the PV group, suggesting a possible M2 phenotype expression in this region and in this group. However, no other significant difference across the groups was demonstrated in our studies. Previous trials in humans failed to show a significant reduction in interleukin expression during UPV, due to the limited reduction in Power_RS_ induced by the strategy (9, 13, 42). On the one hand, we showed that UPV was associated with a considerable reduction in Power_RS_ on the one side, and a decrease in regional lung inflammation in PET, on the other hand, supported by a trend in lower TSPO expression in posterior lung regions in mRNA analysis. However, interleukin expression, macrophage phenotype, or acute lung injury pathology scores did not significantly differ between study groups. We hypothesized that these results may be the consequence of multiple facts. First, the duration of application may have been insufficient to trigger a significant innate immune response, macrophages being VILI first responders (43). However, Cressoni et al. observed the first visible changes in CT images of animals subjected to experimental VILI after 3 h, while lung cellular hypermetabolism assessed with [^18^F]-fluorodeoxyglucose was present only 90 min after VILI onset (44, 45). Extended exposure to the strategy (>4 h) would have potentially resulted in a greater size effect, detectable in both imaging and pathology. Second, pathology and mRNA assays were performed on a limited number of lung samples harvested in four to six lung regions, which may not have been representative of the true extent and severity of acute lung injury, when compared to the spatial resolution allowed by quantitative PET-CT acquisitions. Consequently, we may have lacked the statistical and analytical power to firmly confirm the association of experimental procedures with pathology findings.

The effect of UPV on acute lung inflammation supports its use in patients with ARDS treated with VV-ECMO. However, multiple UPV strategies have been suggested in the literature, and choosing one against another remains debatable. Among all possibilities, apnea has the potential to drastically reduce Power_RS_ and nullify tidal hyperinflation. In consequence, Sorbo et al. observed a significant reduction in biotrauma with apnea, when compared to bilevel ventilation with VV-ECMO (46). Furthermore, our results also support the potential benefit of using low V_T_ ventilation in patients with ARDS not receiving VV-ECMO, when overinflation is associated with lower baby lung volumes. Such a strategy requires, however, tools capable of quantifying hyperinflation in clinical practice, as its systematic application may not generate clinical benefit (41, 47). Finally, we can assume that lung biotrauma decrease may be associated with systemic biotrauma decrease and could limit multiple organ failure incidence in ARDS (48). However, currently, there is no study to support this hypothesis under UPV strategies.

This study also demonstrates several limitations. First, we observed an imbalance between study groups after ARDS induction, suggesting that experimental ARDS was less severe in UPV animals than in PV animals. However, the aerated lung volume after ARDS induction (i.e., the volume exposed to VILI) was similar in both arms. Furthermore, we adjusted the primary outcome analysis for the severity of experimental ARDS, estimated by regional lung weight at end-expiration after experimental ARDS onset. Second, CT ventilation parameters may have been affected by registration errors, especially for animals with very low V_T_. Third, the TSPO expression documented in this study may be specific to this particular model. Indeed, *in vitro* experiments documented a decrease in TSPO expression after pro-inflammatory stimulation in human macrophages (49, 50). However, in our model, [^11^C](R)-PK11195 lung uptake was associated with macrophages recruitment and M1 phenotype expression in previous experiments (6, 11).

## 5 Conclusion

In an experimental model of ARDS, a UPV strategy with VV-ECMO combining ultra-low V_T_, high PEEP, low RR, and low plateau pressure significantly decreased regional macrophagic lung inflammation quantified in PET compared to conventional protective ventilation. Patients with ARDS and treated with VV-ECMO could benefit from similar UPV strategies, given the fact that our results suggest its beneficial effect on ventilator-induced acute lung inflammation.

## Data availability statement

The raw data supporting the conclusions of this article will be made available by the authors, without undue reservation.

## Ethics statement

The animal study was approved by CELYNE, Lyon, reference number 27652/2020101216343732. The study was conducted in accordance with the local legislation and institutional requirements.

## Author contributions

GD: Writing – original draft. FD: Writing – review & editing. SL: Writing – review & editing. MO: Writing – review & editing. ER: Writing – review & editing. WM: Writing – review & editing. NB: Writing – review & editing. J-CR: Writing – original draft, Writing – review & editing. LB: Writing – original draft, Writing – review & editing.
