# Supplementary material for: Pulmonary inflammation decreases with ultra-protective ventilation in experimental ARDS under VV-ECMO: a positron emission tomography study
Source: Front Med (Lausanne). 2024 Feb 20;11:1338602. doi: 10.3389/fmed.2024.1338602 (PMC10912585; doi:10.3389/fmed.2024.1338602)
Supplement: Supplementary file 1 [file Data_Sheet_1.docx]

Supplementary Data

[1 Supplementary Materials and Methods 2](#_Toc154745260)

[1.1 Animal conditioning 2](#_Toc154745261)

[1.2 Experimental ARDS 2](#_Toc154745262)

[1.3 VV ECMO settings 3](#_Toc154745263)

[1.4 Respiratory mechanics 3](#_Toc154745264)

[1.5 CT imaging protocol 4](#_Toc154745265)

[*1.6* *Quantitative lung CT analysis* 4](#_Toc154745266)

[*1.7* [^11^C](R)‐PK11195 PET acquisition protocol 5](#_Toc154745267)

[1.8 Quantification of [^11^C](R)‐PK11195 lung uptake and study primary outcome 6](#_Toc154745268)

[*1.9* Nanostring mRNA multiplex assay 7](#_Toc154745269)

[1.10 Pathology analysis 8](#_Toc154745270)

[2 Supplementary Results 9](#_Toc154745271)

[2.1 Animals and conditioning 9](#_Toc154745272)

[2.2 Effect of UPV on the other 3TCM model parameters at T3 9](#_Toc154745273)

[3 Supplementary Tables 10](#_Toc154745274)

[4 Supplementary Figures 12](#_Toc154745275)

[5 References 21](#_Toc154745276)

# Supplementary Materials and Methods

## Animal conditioning

Animal conditioning was carried out under mechanical ventilation, continuous general anesthesia (propofol 7 mg.kg^‐1^.h^‐1^ and fentanyl 4 µg.kg^‐1^.h^‐1^) and neuromuscular blockade (cisatracrium 1 mg.kg^‐1^.h^‐1^), after prior intramuscular bolus premedication with xylazine 0.7 mg.kg^‐1^, ketamine 2 mg.kg^‐1^ and droperidol 0.2 mg.kg^‐1^.

The following equipment was placed in all animals: 1- a tracheal tube introduced via median surgical tracheotomy, secured hermetically and connected to an eXtend ventilator (Taema, Air Liquide, France) or a Servo-I ventilator (Maquet, Germany) ; 2- a central venous catheter surgically inserted in the left internal jugular (used for drugs’ administration and radiotracer injection); 3- an arterial catheter placed in the carotid artery for continuous hemodynamic monitoring; 4- a pulmonary artery catheter also hosted in the left jugular vein (used to measure the PET radiotracer input function in the pulmonary artery and to measure cardiac output by transpulmonary termodilution). We also inserted an esophageal balloon (Marquat Gbm, France) to allow monitoring of esophageal pressures (P_ES_). The presence of cardiac artifacts, the absence of transmission of gentle epigastric compressions, and adequate correlation of airway pressure (P_AW_) to esophageal pressure (P_ES_) during an occlusion test (quality control criteria: 1.0 ± 0.2 P_ES_ to P_AW_ regression slope, determination coefficient R^2^ > 0.9) were assessed to confirm the correct balloon position and the proper non‐stress volume (1,2).

## Experimental ARDS

Experimental ARDS was induced after animal conditioning, by the intra‐tracheal instillation of a maximum of 5 aliquots of 0.1 ml.kg^-1^  BW of 0.1 M chlorohydric acid (maximum total body weight dose 0.5 ml.kg^‐1^ BW), to reach a S_p_O_2_ < 88% with a F_i_O_2_ of 1 (3). Experimental ARDS was confirmed once the PaO_2_/FiO_2_ ratio was < 150 mmHg 30 minutes after the last aliquot instillation (T2). Arterial blood gas were analysed on a ABL800 FLEX analyzer (Radiometer, France).

## VV ECMO settings

Animals randomized to the UPV strategy received treatment with VV-ECMO. Animal isovolumic canulation was performed before experimental ARDS induction for logistic reasons. HLS cannulas (Maquet, Germany) were positioned in the right jugular vein (15 cm/19 Fr) and in the right femoral vein (50 cm/23 Fr) after surgical dissection. Correct cannula position was confirmed on a CT scout acquisition. We used Rotaflow (Getinge, Germany) or Xenios (Xenios, Fresenius Medical Care, Heilbronn, Germany), with Alone (Euroset, Italy), PLS and Quadrox-iD pediatric (Maquet, Germany) or Novalung (Xenios, Fresenius Medical Care, Germany) membranes and a circuit heater unit (HU 35, Maquet, USA). The heater unit was set at a temperature of 37°C. In case of VV-ECMO pump dysfunction, intravenous fluid therapy (Ringer Lactate solution, 500 ml) was administrated. In case of low mean arterial pressure < 70 mmHg without VV-ECMO dysfunction, intravenous norepinephrine was administered continuously and adjusted to maintain the mean arterial pressure between 70 and 80 mmHg.

## Respiratory mechanics

All airway and esophageal pressures and gas flow were acquired and analysed with the Acqknowledge® software (Biopac Systems, CA, USA). Throughout the protocol, we regularly monitored the following variables: RR, V_T_, total PEEP (P_AW,EE_), airway plateau pressure (P_AW,EI_) and esophageal pressures. At the end of a 3-second expiratory pause, P_AW,EE_ and end‐expiratory P_ES_ (P_ES,EE_) were measured, and P_AW,EI_ and end‐inspiratory P_ES_ (P_ES,EI_) at the end of a 3‐sec inspiratory pause (4). From these, we determined the difference between end‐inspiratory and end‐expiratory airway and esophageal pressures (∆P_AW_ and ∆P_ES_), respectively. Respiratory system elastance (El_RS_), chest wall elastance (El_CW_), and lung elastance (El_L_), were calculated respectively by dividing ∆P_AW_, ∆P_ES_ and the difference between them by V_T_. Lung end-inspiratory transpulmonary driving pressures P_L,EI_ were estimated using the ratio of El_L_ to El_RS_ (5). Mechanical power delivered to the respiratory system (Power_RS_, in J.min^-1^) was defined using the equation of motion (6).

${Power}_{RS}=0.098 \cdot RR\cdot\left[ V_{T}^{2}\cdot{El}_{RS}\cdot\frac{1}{2}+V_{T}\cdot R_{aw}\cdot F+V_{T}\cdot PEEP \right]$ Eq. 1

with *R_aw_*: airway resistances with $R_{aw}= \left[ P_{AW,MAX}-P_{AW,EI} \right]/F$ Eq. 2

*RR*: respiratory rate

*P_AW, MAX_* : peak airway pressure at inspiration

*F*: gas flow

## CT imaging protocol

Longitudinal field‐of‐view (FOV) is comprised between caudal lung extremity and lung apex. CT settings were the following: voltage 120 keV, intensity 66 mAs, pitch 0.6, FOV diameter 780 mm, image reconstruction with a B31f smooth filter, slice thickness 1 mm, matrix size 512×512, and pixel size 0.57×0.57 mm.

## *Quantitative lung CT analysis*

We used MatLab (Natick, Massachusetts, USA) to assess aeration and CT volume in each ROI determined above (whole lung and 8 lung levels). Voxel gas fraction was estimated with the voxel CT number in Hounsfield units (HU) (7). ROI lung weight was estimated by the product of the ROI volume by its non‐gas fraction (1 – F_GAS_), considering a lung tissue density of 1. We identified the following aeration compartments on each ROI: nonaerated (‐100 to +100 HU), poorly aerated (‐500 to ‐101 HU), normally aerated (‐900 to ‐501 HU), and hyperinflated (‐1000 to ‐901 HU)] (8). The regional gas volume at end‐expiration (EELV), end‐inspiration (EILV), and at FRC (V_FRC_) corresponded to the ROI volume multiplied by its gas fraction F_GAZ_ (9). The following CT-derived volumes were normalised to the animal BW (in kg) and determined as follows:

- CT‐derived V_T_ (V_T,CT_): difference between EILV and EELV.
- PEEP‐related gas volume (V_PEEP_): difference between EELV and V_FRC_
- Tidal lung recruitment and PEEP‐related lung recruitment: the change in the non‐aerated compartment weight (-100 to +100 HU) associated with V_T_ and PEEP application and converted to ml using the end‐expiratory and FRC gas fractions (on the range -1000 to -101 HU), respectively (10).
- Tidal hyperinflation: difference in hyperinflated volumes (-1000 to -901 HU) at end‐inspiration and end‐expiration.
- Dynamic strain: ratio of V_T,CT_ to EELV; and static strain: ratio of V_PEEP_ to V_FRC_, corrected for the effects of tidal and PEEP recruitment, respectively (11).
- Regional respiratory system compliance at end-expiration (C_RS,PEEP_): ratio of regional V_PEEP_ to [total PEEP – P_atmo_] (with P_atmo_ = 0 cmH_2_O).

## [^11^C](R)‐PK11195 PET acquisition protocol

A 60 min PET acquisition with [^11^C](R)-PK11195 was performed at T3 in all animals. The 216-mm longitudinal FOV extended from the caudal lung extremity to its apex. PET frames were reconstructed in 3D volumes of 112 slices (thickness 2mm) and 128×128 matrices (2.1×2.1 mm pixel size), in dynamic series of 18 frames (5×12 sec, 4×60 sec, 7×300 sec, and 2×600 sec). We corrected the signal for random coincidences, attenuation, scatter and ^11^C isotope decay. To obtain regional PET time-activity curves (TAC), ROI masks were segmented on the lung transmission CT as described in “CT imaging protocol”. The dose of [^11^C](R)‐PK11195 injected was not significantly different among experimental groups (4.7 [4.5-5.0] MBq.kg^-1^ BW in UPV animals vs. 5.0 [4.6-5.1] MBq.kg^-1^ BW in the control group, p=0.73). The non-invasive corrected input function was unaffected by the use of VV ECMO in the UPV group (Supplementary Figure 7).

## Quantification of [^11^C](R)‐PK11195 lung uptake and study primary outcome

The 3TCM model requires the determination of six constants: the fraction of whole blood volume in the ROI (F_WB_), the entry rate constant from blood to tissue corrected for the regional tissue fraction estimated on the CT (*K_1_* in ml of plasma.min^‐1^.ml^‐1^.g^-1^ of lung), the backward rate constant of transfer from tissue to blood (*k_2_* in min^‐1^), the rate constant representing [^11^C](R)‐PK11195 binding to the TSPO receptor (*k_3_* in min^‐1^), the rate constant corresponding to its dissociation from its receptor (*k_4_* in min^‐1^), and the rate constant of irreversible uptake by the non‐specific compartment (*k_5_* in min^‐1^). *K_1_* to *k_2_* ratio expresses the parent tracer’s net influx from the plasma pool to tissue. The 2TCM was similar to the 3TCM, with the exception of the compartment defined above (described by the *k_5_* rate constant, Supplementary figure 8).

In each lung ROI, resolution of both models (2TCM and 3TCM) was performed using the parent plasma non‐invasive input function C_PK_(t), and the lung ROI time-activity curve (TAC) C_ROI_(t) as input data, by mean of weighted non‐linear least‐square resolution. C_PK_(t) was the parent plasma non‐invasive input function and corresponded to the whole blood radioactivity measured in the pulmonary artery on PET images, corrected for spill‐over, partial volume, plasma fraction, metabolite fraction, and regional tissue arrival delay, as previously described (12). Finally , to select which model best described the lung TAC of a ROI, we applied a 2‐step model selection algorithm which led to the determination of ROI’s BP_ND_ value (Supplementary Figure 9) (12).

## Nanostring mRNA multiplex assay

At the end of the protocol, animals were euthanized by the intravenous injection of 180 mg.kg^-1^ of pentobarbital followed by the disconnection from the ventilator. The left lung was divided in 4 regions (antero-cephalic, antero-caudal, postero-cephalic, postero-caudal). We sampled one cubic centimeter tissue fragment in each region. Lungs samples were snap frozen in liquid nitrogen and kept at -80° for long-term storage. Fifty 20 μm frozen sections were then made from the sample using a cryostat (Leica Biosystems CM3050S, Wetzlar, Germany). We used RNeasy Blood Mini Kit (Qiagen, Hilden, Germany) to extract RNA from frozen sections. RNA quantity and quality were determined using Nanodrop (Thermofisher Scientific, MA, USA). The NanoString nCounter® technology (NanoString Technologies, Seattle, WA, USA), a hybridization-based multiplex assay characterized by its amplification-free step, was used for mRNA detection of a multigene custom panel designed with genes known to be crucial to the macrophages/neutrophils immune response (13). Seven mRNAs of interest made up the panel [i.e., TSPO, the target of [^11^C](R)-PK11195 (accession number NM_213753.1), CD68, a protein expressed on the surface of monocytes and macrophages (NM_001291776.1), FCGR3A/B (CD16a/b), a mRNA produced by natural killer cells and neutrophils (NM_214391.2), CD84, a protein expressed on the surface of M1 macrophages (XM_001928464.6), and CD163, a protein expressed on the surface of M2 macrophages (NM_213976.1)], IL10, a cytokine promoting M2 phenotype (NM_214041.1), IL6, a cytokine promoting M1 phenotype (NM 001252429.1)] and 3 housekeeping mRNAs [i.e., TBP (XM_013991786.1), SDHA (XM_021076930.1) and RPL4 (XM_005659862.2)] (14).

Samples’ preparation was performed according to the manufacturer: 1. 100 ng of RNA were hybridized to the probes at 67°C for 18 hours using a thermocycler; 2. excess probes were then removed; 3. samples were loaded into the nCounter Prep Station (NanoString Technologies, Seattle, WA, USA) for purification and immobilization on to the internal surface of a sample cartridge for 2-3 hours. The sample cartridge was then transferred and imaged on the nCounter Digital Analyzer (NanoString Technologies, Seattle, WA, USA) where colour codes were counted and tabulated for the selected genes. Data generated were treated and normalized using nSolver analysis software (version 4.0, NanoString technologies, Seattle, WA, USA). Each sample was analysed in a separate multiplexed reaction, each including 8 negative probes and 6 serial concentrations of positive control probes. Negative control analysis was performed to determine the background for each sample. The internal positive controls allowed correction of a potential source of variation associated with the technical platform and was performed as follows. First, we determined in all samples the background level as the median +2 SD across the six negative probe counts (obtained threshold: 21.4). Then, every sample under the background level was fixed to this value. Next, we calculated for each sample the geometric mean of the positive probe counts. The scaling factor for a sample was the ratio of the geometric mean of the sample and the average across all geometric means. For each sample, we divided all gene counts by the corresponding scaling factor. To normalize for differences in RNA input we used the same method as in the positive control normalization, except that geometric means were calculated over the 3 housekeeping genes. For the analysis, antero-cephalic and antero-caudal samples values were pooled under the term “Anterior lung region”; postero-cephalic and postero-caudal samples values under the term “Posterior lung region”.

## Pathology analysis

After lung extractions, the right one was divided into an anterior region and a posterior region. In each region, 3 samples of a cubic centimetre were sampled in the cephalic, the median and caudal tiers of the region, respectively (resulting in 6 samples per animal). Samples were prepared for pathology analysis with hematoxylin and eosin staining.

# Supplementary Results

## Animals and conditioning

There was a trend towards a lower weight in animals in PV group than in UPV group (36 [IQR, 33-38] kg in UPV group and 30 [29-31] kg, p=0.16). There was no statistical difference in the amount of chlorohydric acid instilled (144 [114-150] ml in UPV group and 135 [120-145] ml in PV group, p=0.49)

## Effect of UPV on the other 3TCM model parameters at T3

Lung perfusion was lower in anterior lung regions in both groups (Supplementary Figure 5). We hypothesize that the redistribution to posterior regions of regional blood flow as a consequence of occurrence of hyperinflation in the same lung regions. Indeed, hyperinflation may generate pulmonary capillaries compression and increase ventilation-perfusion mismatch. We hypothesized that the ECMO extracorporeal circuit may trap the radiotracer due to its lipophilic features, and would have increased the estimated concentration of tracer in the non-specifically and irreversibly trapped C_ns_(t). However, we made the opposite observation of lower *k_5_* values (closer to 0 min^-1^) in this group. This might be explained by lower bio-availaibility of the radiotracer in UPV animals, which have been less prone to saturate this compartment. However, despite the expected increase in the distribution volume of the radiotracer in this group, injected radiotracer doses were similar in both groups, *K_1_*/*k_2_* ratio were also similar across the groups and non-invasive input functions measured downstream in the pulmonary artery were comparable between controls and UPV animals (Supplementary Figure 1).

# Supplementary Tables

| **Supplementary Table 1. Biology over time** | | | | | |  |
| --- | --- | --- | --- | --- | --- | --- |
|  | **Study group** | |  |  |  | |
| Variables | PV  n=5 | UPV  n=5 | Effect of group, p | Effect of time, p | Group × time, p | |
| PaO_2_/FiO_2_ ratio |  |  | 0.99 | 0.87 | 0.17 | |
| After lung injury | 80 [69-95] | 87 [69-133] |  |  |  | |
| 3-4 hours after injury | 77 [74.5-126] | 74 [58.5-96] |  |  |  | |
| pH |  |  | 0.16 | 0.12 | 0.33 | |
| After lung injury | 7.32 [7.29-7.32] | 7.24 [7.17-7.35] |  |  |  | |
| 3-4 hours after injury | 7.27 [7.26-7.28] | 7.14 [7.13-7.21] |  |  |  | |
| PCO_2_, mmHg |  |  | 0.61 | 0.17 | 0.99 | |
| After lung injury | 59 [58-60] | 66 [41-68] |  |  |  | |
| 3-4 hours after injury | 69 [65-75] | 61 [57-73] |  |  |  | |
| Lactates, mmol.L^-1^ |  |  | 0.15 | 0.47 | 0.22 | |
| After lung injury | 1.1 [0.9-1.2] | 2.2 [1.5-2.3] |  |  |  | |
| At the end of protocol | 0.7 [0.6-1.5] | 2.8 [1.9-5.2] |  |  |  | |
| Haemoglobin, g.L^-1^ |  |  | 0.84 | 0 .02 | 0.06 | |
| After lung injury | 99 [78-102] | 94 [83-97] |  |  |  | |
| 3-4 hours after injury | 93 [93-113] | 103 [93-107] |  |  |  | |

Values are median with interquartile range. Study time points were after experimental ARDS onset and 3h to 4h later (i.e. 1h or just before protocol end). Mixed effects linear regressions with study group and study time point as independent variables, and animal identification number as random effect, were used to compare variables at different study times and in the two groups. Interaction of time study with the group was systematically checked for. If no interaction was identified, the p value of the group effect and the study time effect are given respectively. In case of significant interaction, a pairwise post-hoc multiple comparison was performed to compare groups at each time point on the one side and compare the 2 study times in each group, on the other. Multiple comparisons were adjusted for α inflation using the Benjamini and Hochberg method.

UPV: ultra-protective ventilation; PV: protective ventilation; PaO_2_ : arterial O_2_ partial pressure, PaCO_2_ : arterial CO_2_ partial pressure, PaO_2_/FiO_2_: ratio of arterial O_2_ partial pressure to O_2_ inspired fraction.

| **Supplementary Table 2. Association of regional CT parameters with regional BP_ND_ in univariate analysis** | | | |  |
| --- | --- | --- | --- | --- |
| **Regional variables** (80 measurements) | **Regional BP_ND_**  β±SE | **p value** | **Variables included in multivariate analysis** | |
| Lung levels (from 1 [most anterior] to 3 [most posterior]) | -0.17 ± 0.07 | 0.02 | **Included^*^** | |
| Lung tissue weight at end-expiration, per 1 g.kg^-1^ BW increase | -0.12 ± 0.12 | 0.33 | *nr* | |
| EELV, per 1 ml.kg^-1^ BW increase | -0.26 ± 0.07 | <0.01 | *mc* ^a^ | |
| End-expiratory hyperinflated volume, per 1 ml.kg^-1^ BW increase | -0.24 ± 0.13 | 0.06 | *nr* | |
| Regional C_RS,PEEP_, per 1 ml.cmH_2_O.kg^-1^ BW increase | -0.25 ± 0.07 | <0.01 | **Included^a,*^** | |
| PEEP-related alveolar recruitment, per 1 ml.kg^-1^ BW increase | -0.02 ± 0.09 | 0.76 | *nr* | |
| Static strain, per 1 unit | -0.10 ± 0.07 | 0.22 | *nr* | |
| V_T,CT_, per 1 ml.kg^-1^ BW increase | 0.08 ± 0.09 | 0.41 | *mc* ^b^ | |
| Tidal recruitment, per 1 ml.kg^-1^ BW increase | 0.07 ± 0.09 | 0.47 | *nr* | |
| Tidal hyperinflation, per 1 ml.kg^-1^ BW increase | -0.46 ± 0.11 | <0.01 | **Included^b,*^** | |
| Dynamic strain, per 1 unit | 0.16 ± 0.09 | 0.08 | *nr* | |

“Anterior lung levels” regroups lung regions 1 and 2, “Median lung levels” regroups lung region 3 to 6 and “Posterior lung levels” regroups lung region 7 and 8. Variables were included in the multivariate analysis because of their physiological relevance after seeking for collinearity and interactions. Variables with a Pearson correlation coefficient > 0.8 and/or a VIF > 3 were excluded from the multivariate model for collinearity. Variables were excluded from the model in a forward and backward stepwise manner, if it did not improve the goodness-of-fit of the model (based on ANOVA of full models with and without the variable). End-inspiratory CT were missing in two animals in the UPV group. Missing data in variables derived from these CT were handled using multiple imputations (using a “predictive mean matching” procedure). We retained the interaction between lung levels, regional C_RS,PEEP_ and tidal hyperinflation in the final model. The significance of this interaction was evaluated using bootstrap with 500 simulations in each datasets (n=20) generated after imputation of missing data. The final model marginal R2 was 0.34 [0.32-0.36] and the conditional R2 was 0.43 [0.42-0.45]. Model residuals were checked graphically for normality.

^a^: multicollinearity between EELV and regional C_RS,PEEP_; ^b^: multicollinearity between tidal hyperinflation and V_T,CT_; **^*^** : interaction between lung levels, regional C_RS,PEEP_ and tidal hyperinflation; β : model coefficient; BP_ND_ : [^11^C](R)‐PK11195 binding potential; BW: body weight ; EELV: end-expiratory lung volume; Regional C_RS,PEEP_ : regional respiratory system compliance at PEEP; PEEP: positive end-expiratory pressure; SE : standard error; VIF: variance inflation factor; V_T,CT_: computerized tomography-derived tidal volume; *mc*: variable not included in multivariate analysis because of (multi)collinearity; *nr*: included in multivariate model, but not retained in the final model after stepwise selection.

#
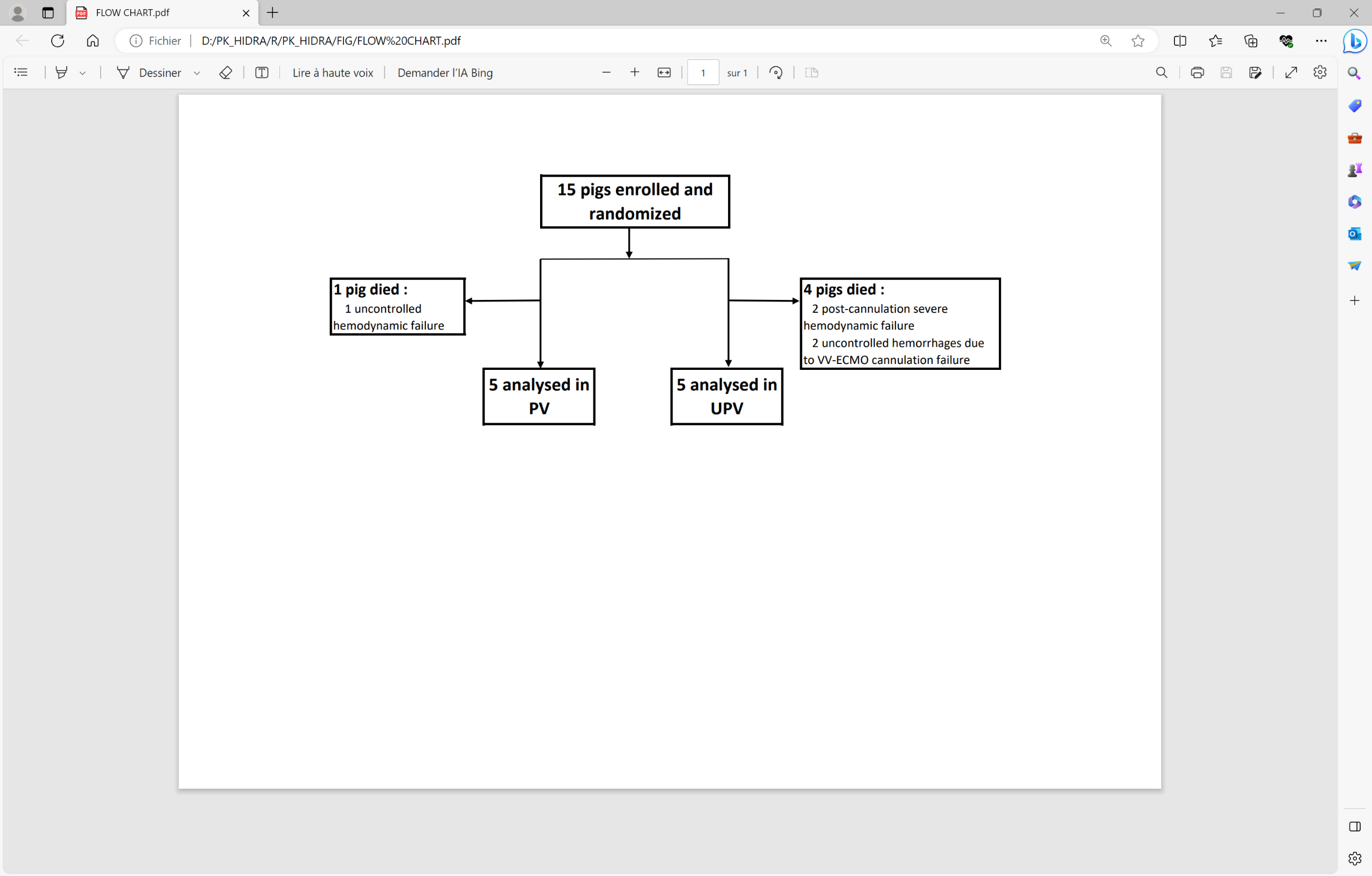
Supplementary Figures

**Supplementary Figure 1. Flow Chart**

The figure shows the study flow chart.

PV : protective ventilation strategy ; UPV: ultra-protective ventilation strategy ; VV-ECMO : veno-venous extracorporeal membrane oxygenation.


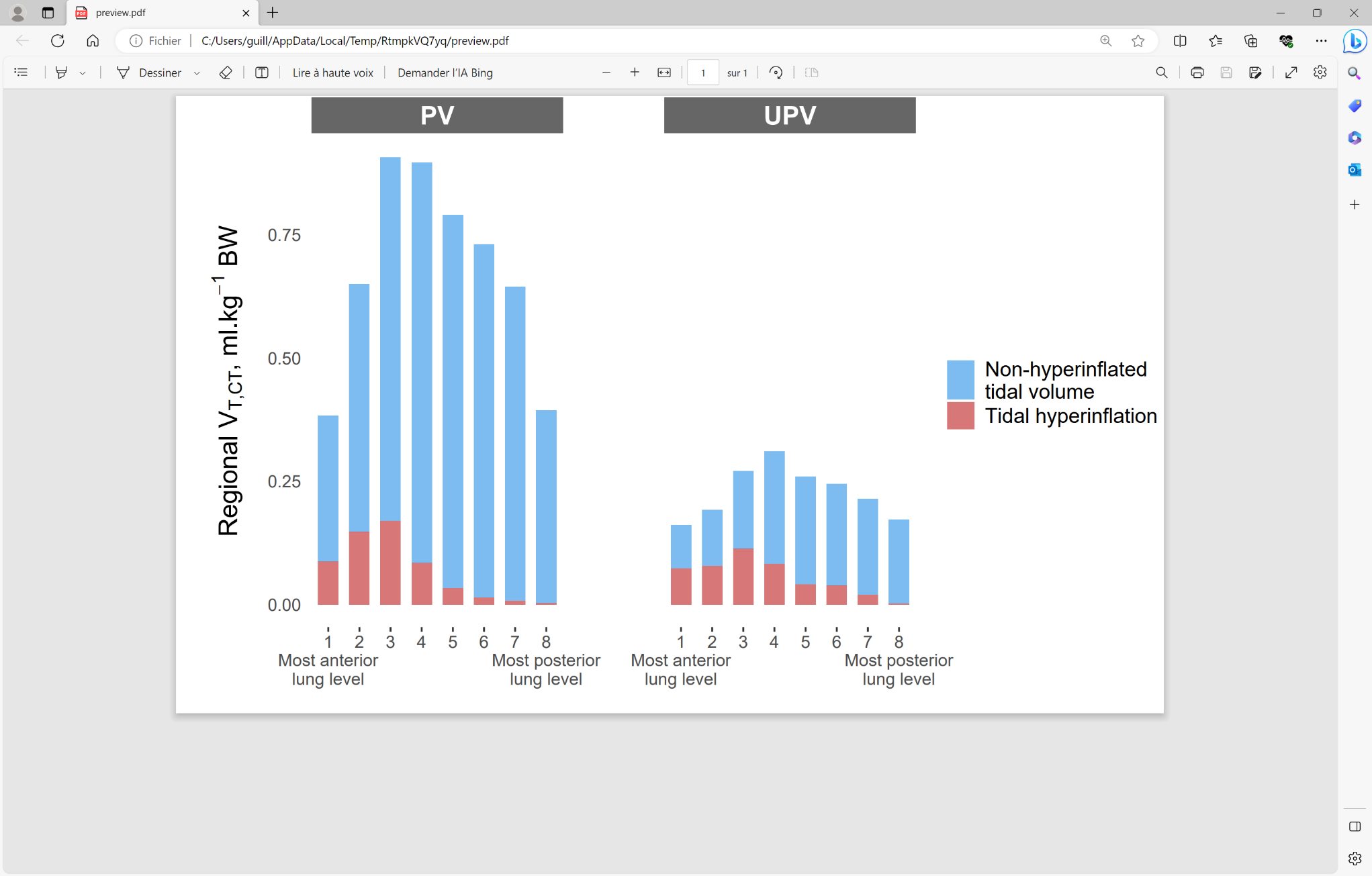
**Supplementary Figure 2. Hyperinflation distribution across study groups and lung regions**

The figure shows tidal hyperinflation proportion in tidal volume across lung regions (from 1 [i.e., the most anterior] to 8 [i.e., the most posterior]) and between the two study groups. The whole bar plots represent tidal volume, the red part tidal hyperinflation and the blue the non-hyperinflated tidal volume.

PV: protective ventilation ; UPV: ultra-protective ventilation ; BW : body weight


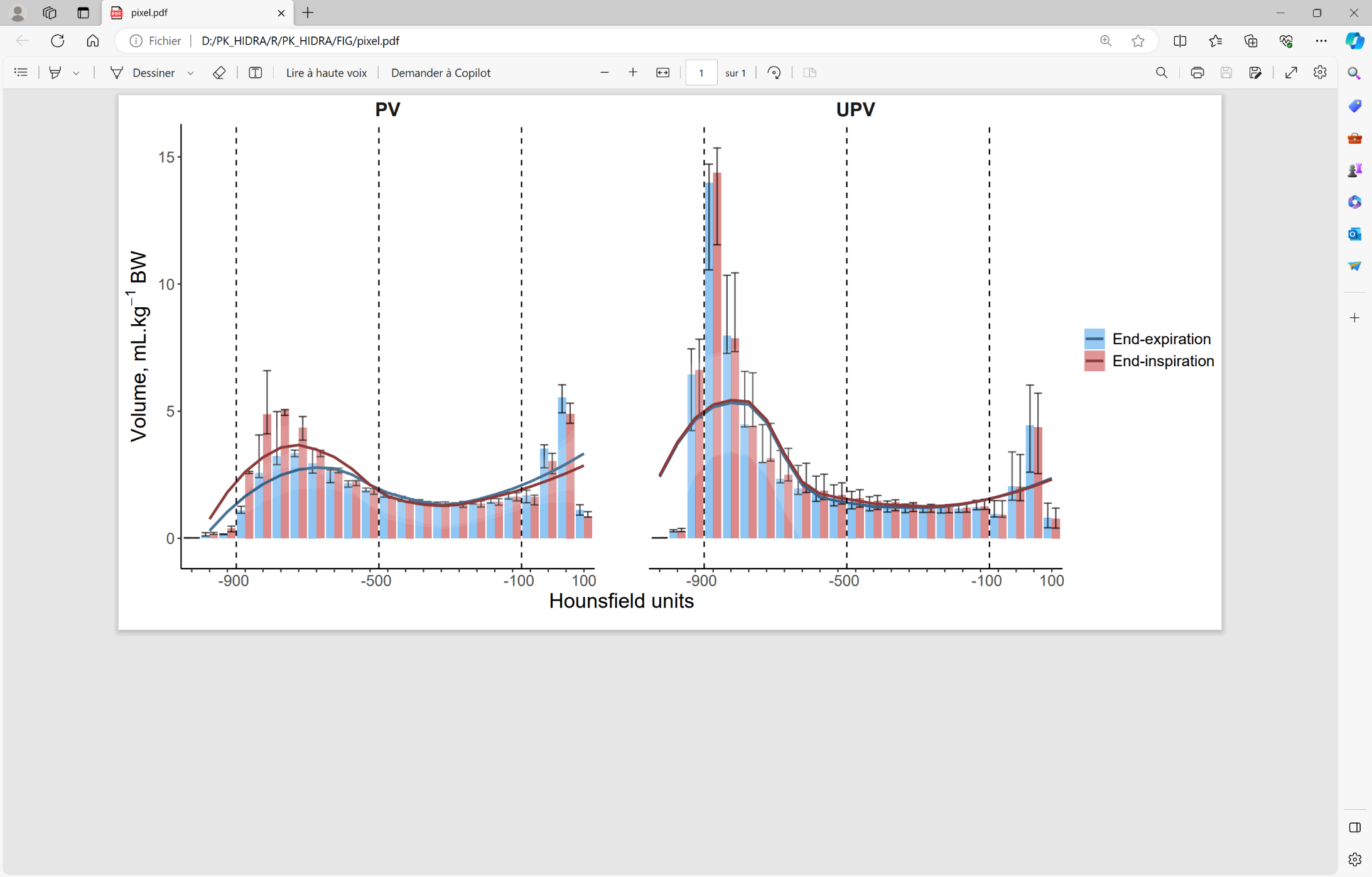
**Supplementary Figure 3. CT imaging compartments volume across groups and respiratory times**

The figure shows CT lung compartment volume based on pixel grey level in Hounsfield units (HU). 23 bins of 50 HU have been defined. Columns represent each median lung compartment volume with their error bar representing interquartile, across study groups and respiratory times. Dashed vertical lines represent limits of non-aerated lung volume [-100:100], poorly-aerated lung volume [-100:-500], normally-aerated lung volume [-500:-900] and hyperinflated lung volume [-900:-1024]. Colour lines are graphic LOESS regression representations.

PV : protective ventilation, UPV: ultra-protective ventilation, CT : computed tomography, BW : body weight


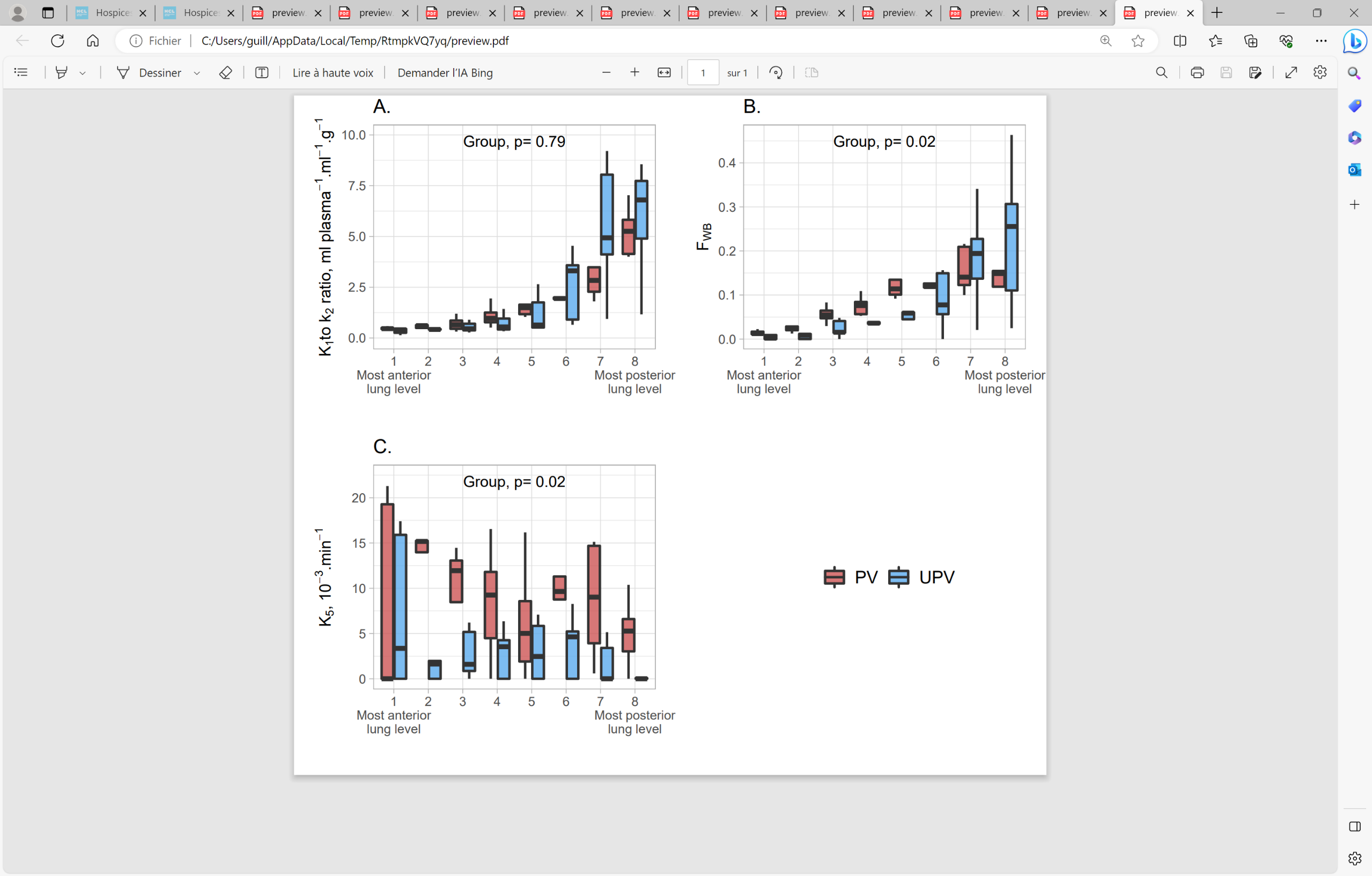
**Supplementary Figure 4.  Regional [^11^C](R)‐PK11195 kinetic model parameter estimates at T3**

The figure shows the regional K_1_ to k_2_ ratio corrected for the regional tissue fraction, whole blood fraction F_WB_ and k_5_ in the 8 lung regions in UPV group (blue boxplots) and in the control group (red boxplots). The p value evaluates the association between the studied parameter and the interaction of Group x lung Level (including a quadratic factor or an exponential factor, if significant). In case of a non-significant interaction term, the p value of the independent effect of Group is given. F_WB_: regional whole blood fraction.


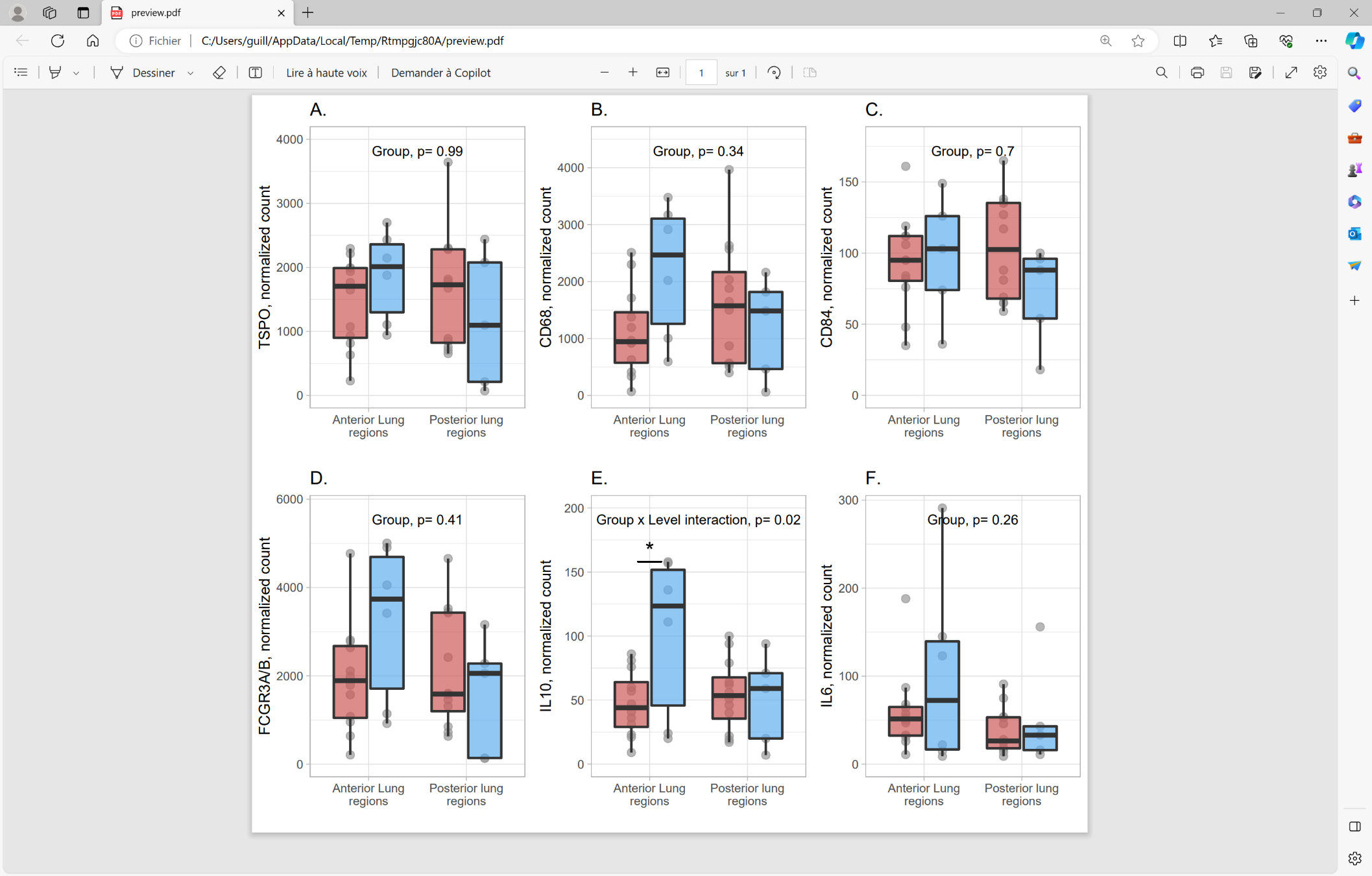


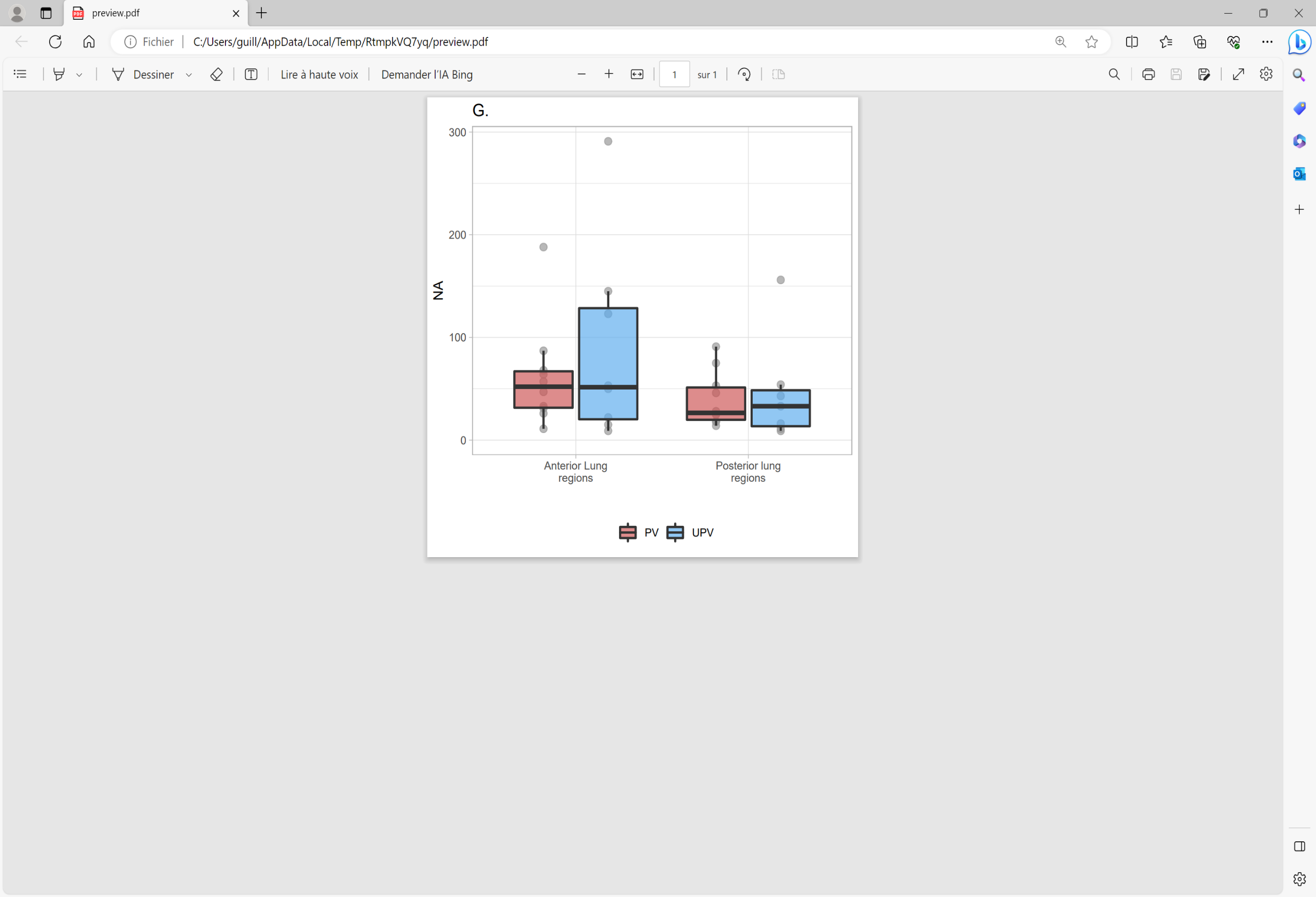


**Supplementary Figure 5. Lung mRNA assays in both study groups**

Panels A to F show the results of the mRNA multi-gene panel performed with the Nanostring technology in two lung regions (anterior and posterior), showing the quantity of mRNA (expressed in normalized counts) of TSPO (A), CD68 (B), CD84 (C), FCGR3A/B (D), IL10 (E) and IL6 (F). The panels show the individual value observed in each region, and corresponding boxplots. The p value evaluates the association between the studied parameter and the interaction of Group x lung Level. Significance of the multivariate model was evaluated using bootstrap with 500 simulations. In case of a significant interaction term, a comparison of the marginal means evaluates the difference between groups in each lung region. On the other hand, the p value of the independent effect of Group is given.

* p<0.05; PV: protective ventilation; UPV: ultra-protective ventilation strategy; TSPO: translocator protein.


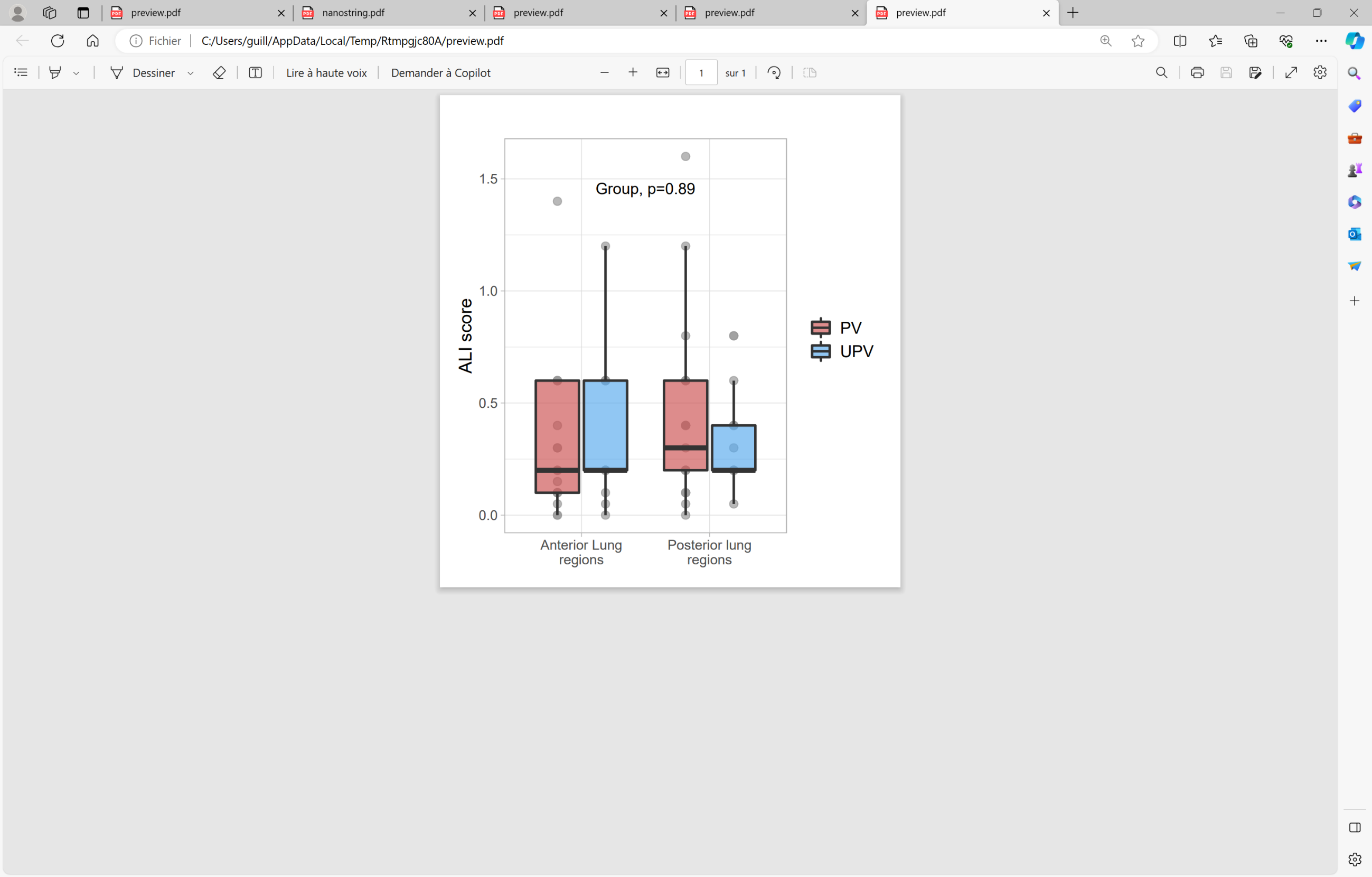


**Supplementary Figure 6. Pathology study in both groups**

The figure shows the ALI score evaluated by a pathologist blinded to the intervention. The p value evaluates the association between the ALI score and the interaction of Group x lung Level. In case of a non-significant interaction term, p value evaluates the association between the study group and the ALI score.

ALI score: acute lung injury score; UPV: ultra-protective ventilation strategy.


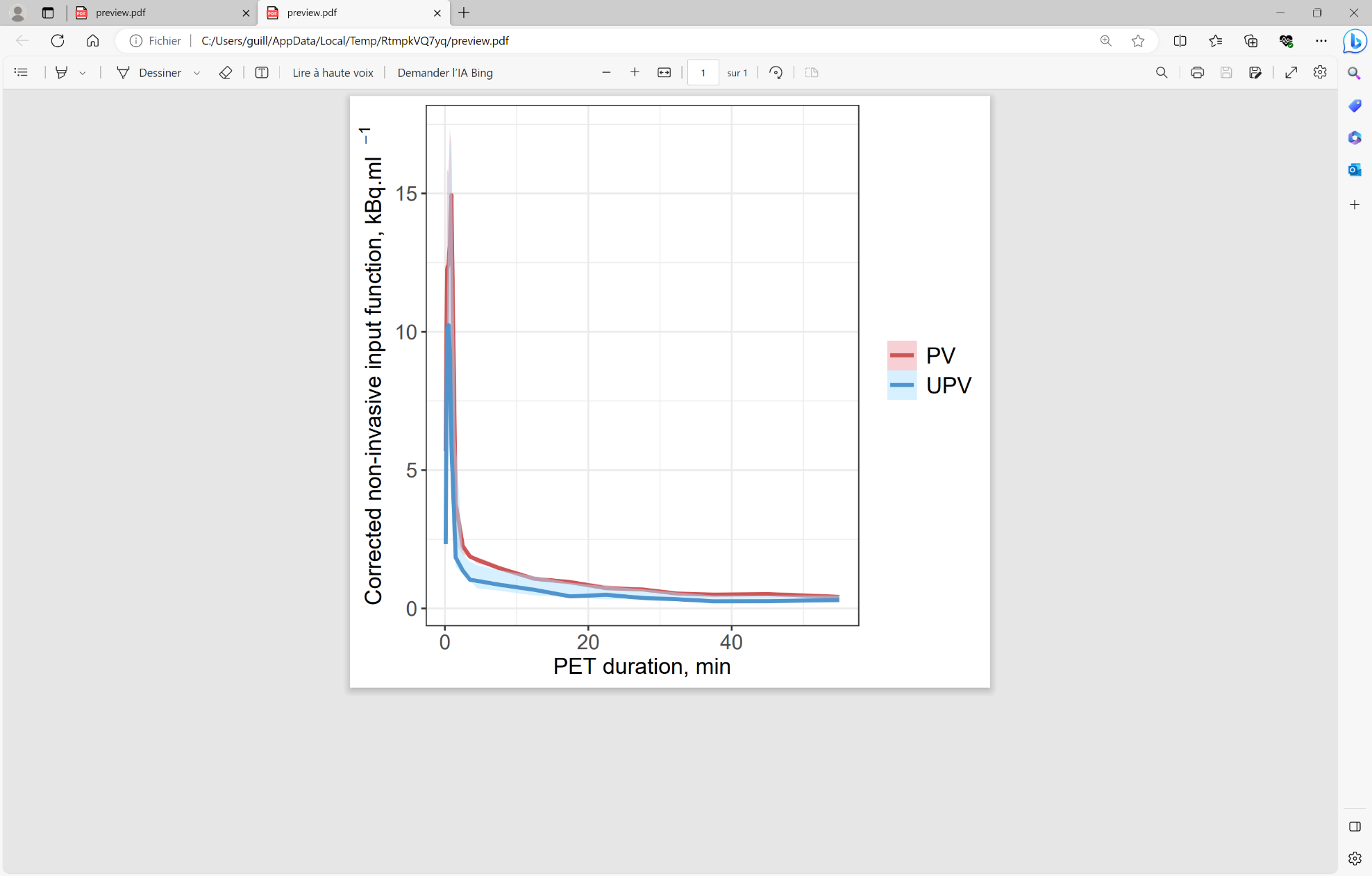


**Supplementary Figure 7. Non-invasive input function**

The figure shows the non-invasive input function corrected for spill‐over, partial volume, plasma fraction, metabolite fraction, and regional tissue arrival delay between study groups (as the median values and their interquartile range).

kBq: kilobecquerel; PV: protective ventilation; UPV: ultra-protective ventilation strategy; PET: positron emission tomography.


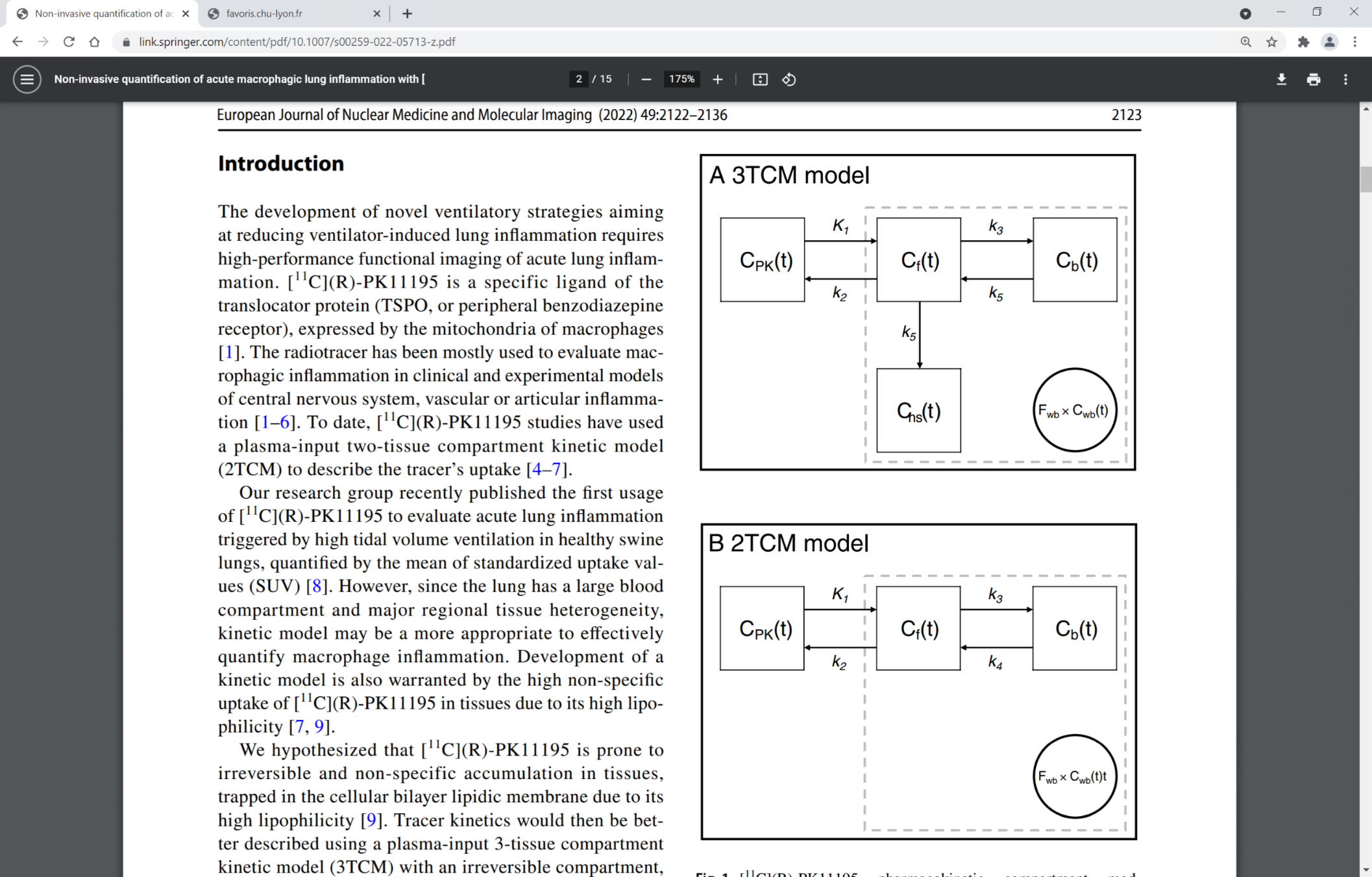


**Supplementary Figure 8. Compartment kinetic models**

[^11^C](R)-PK11195 pharmacokinetic compartment models (12). Panel A shows the 3-compartment kinetic model of [^11^C](R)- PK11195 uptake in the lung, using a plasma input function C_PK_(t). Cf (t) is the concentration of free parent tracer in lung tissue over time (compartment #1), C_b_(t) is the concentration of parent compound bound to the TSPO receptor in the ROI over time (compartment #2), and C_ns_(t) is the concentration of tracer non-specifically and irreversibly trapped in the tissue over time (compartment #3). F_WB_ × C_wb_(t) represents the fractional concentration of whole blood present in the ROI over time. The lung ROI is represented by the dashed gray line. Rate constants kx are associated with arrows showing the direction of the kinetic transfer they describe. Panel B shows the two-tissue compartment model (2TCM), with the same design as the model presented in panel A, without the irreversible non-specifc compartment. ROI, region of interest; 2TCM, two-tissue compartment model; 3TCM, three-tissue compartment model; TSPO, translocator protein.

*Reproduced with authorization of Springer Nature*


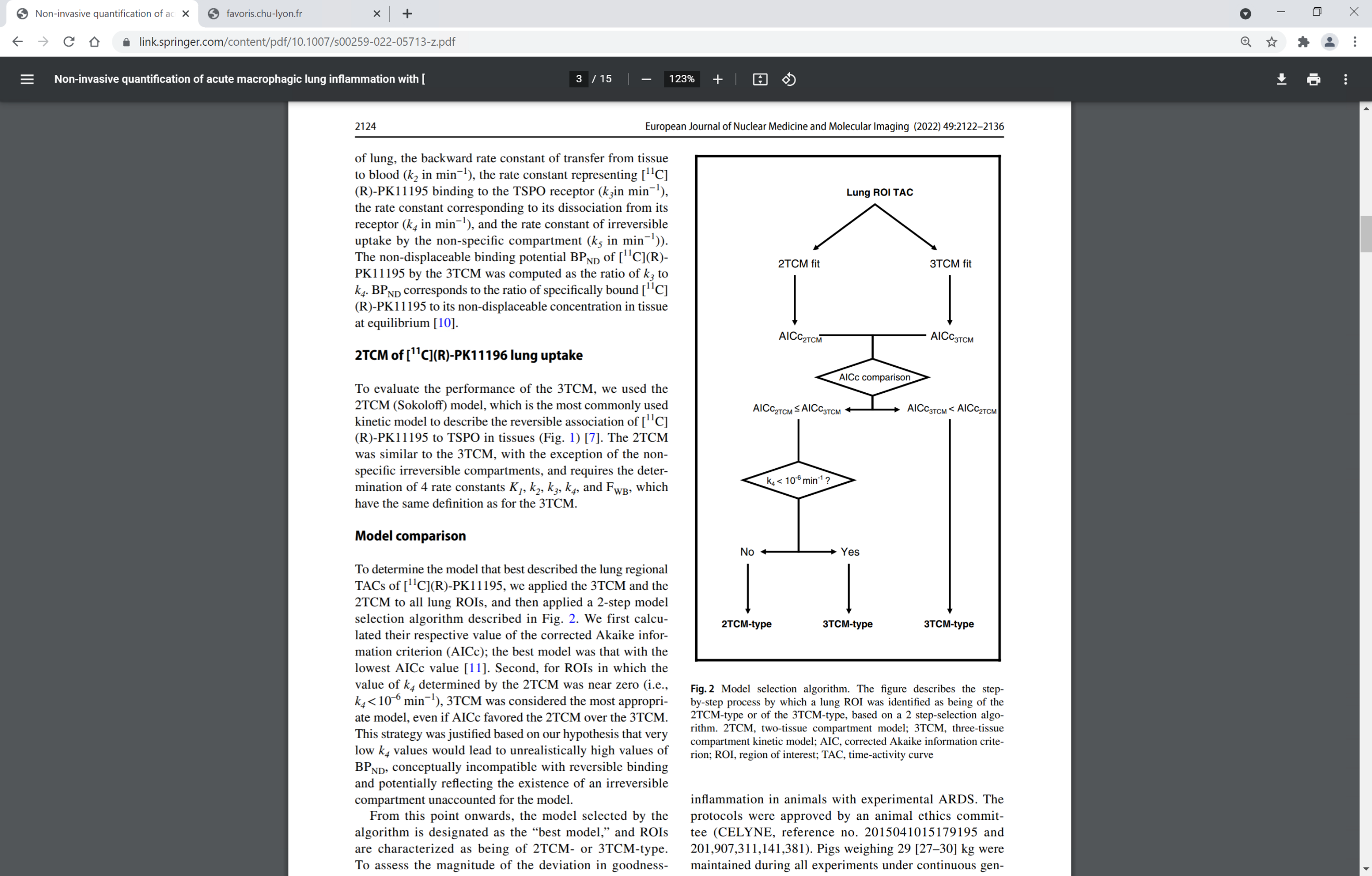


**Supplementary Figure 9. Compartment kinetic model selection**

Model selection algorithm (12). The figure describes the step-by-step process, by which a lung ROI was identified as being of the 2TCM-type or of the 3TCM-type, based on a 2 step-selection algorithm. 2TCM, two-tissue compartment model; 3TCM, three-tissue compartment kinetic model; AIC, corrected Akaike information criterion; ROI, region of interest; TAC, time-activity curve.

*Reproduced with authorization of Springer Nature*.

# References

1. Talmor D, Sarge T, Malhotra A, O’Donnell CR, Ritz R, Lisbon A, Novack V, Loring SH. Mechanical Ventilation Guided by Esophageal Pressure in Acute Lung Injury. *N Engl J Med* (2008) 359:2095–2104. doi: 10.1056/NEJMoa0708638

2. Yoshida T, Amato MBP, Grieco DL, Chen L, Lima CAS, Roldan R, Morais CCA, Gomes S, Costa ELV, Cardoso PFG, et al. Esophageal Manometry and Regional Transpulmonary Pressure in Lung Injury. *Am J Respir Crit Care Med* (2018) 197:1018–1026. doi: 10.1164/rccm.201709-1806OC

3. Matute-Bello G, Downey G, Moore BB, Groshong SD, Matthay MA, Slutsky AS, Kuebler WM, Acute Lung Injury in Animals Study Group. An official American Thoracic Society workshop report: features and measurements of experimental acute lung injury in animals. *Am J Respir Cell Mol Biol* (2011) 44:725–738. doi: 10.1165/rcmb.2009-0210ST

4. Barberis L, Manno E, Guérin C. Effect of end-inspiratory pause duration on plateau pressure in mechanically ventilated patients. *Intensive Care Med* (2003) 29:130–134. doi: 10.1007/s00134-002-1568-z

5. Grieco DL, Chen L, Brochard L. Transpulmonary pressure: importance and limits. *Ann Transl Med* (2017) 5:285. doi: 10.21037/atm.2017.07.22

6. Gattinoni L, Tonetti T, Cressoni M, Cadringher P, Herrmann P, Moerer O, Protti A, Gotti M, Chiurazzi C, Carlesso E, et al. Ventilator-related causes of lung injury: the mechanical power. *Intensive Care Med* (2016) 42:1567–1575. doi: 10.1007/s00134-016-4505-2

7. Gattinoni L, Pesenti A, Bombino M, Baglioni S, Rivolta M, Rossi F, Rossi G, Fumagalli R, Marcolin R, Mascheroni D. Relationships between lung computed tomographic density, gas exchange, and PEEP in acute respiratory failure. *Anesthesiology* (1988) 69:824–832. doi: 10.1097/00000542-198812000-00005

8. Terragni PP, Rosboch G, Tealdi A, Corno E, Menaldo E, Davini O, Gandini G, Herrmann P, Mascia L, Quintel M, et al. Tidal hyperinflation during low tidal volume ventilation in acute respiratory distress syndrome. *Am J Respir Crit Care Med* (2007) 175:160–166. doi: 10.1164/rccm.200607-915OC

9. Gattinoni L, Pelosi P, Crotti S, Valenza F. Effects of positive end-expiratory pressure on regional distribution of tidal volume and recruitment in adult respiratory distress syndrome. *Am J Respir Crit Care Med* (1995) 151:1807–1814. doi: 10.1164/ajrccm.151.6.7767524

10. Paula LF, Wellman TJ, Winkler T, Spieth PM, Güldner A, Venegas JG, Gama de Abreu M, Carvalho AR, Vidal Melo MF. Regional tidal lung strain in mechanically ventilated normal lungs. *J Appl Physiol (1985)* (2016) 121:1335–1347. doi: 10.1152/japplphysiol.00861.2015

11. Bitker L, Costes N, Le Bars D, Lavenne F, Orkisz M, Hernandez Hoyos M, Benzerdjeb N, Devouassoux M, Richard J-C. Noninvasive quantification of macrophagic lung recruitment during experimental ventilation-induced lung injury. *J Appl Physiol (1985)* (2019) 127:546–558. doi: 10.1152/japplphysiol.00825.2018

12. Bitker L, Dhelft F, Lancelot S, Le Bars D, Costes N, Benzerdjeb N, Orkisz M, Richard J-C. Non-invasive quantification of acute macrophagic lung inflammation with [11C](R)-PK11195 using a three-tissue compartment kinetic model in experimental acute respiratory distress syndrome. *Eur J Nucl Med Mol Imaging* (2022) 49:2122–2136. doi: 10.1007/s00259-022-05713-z

13. Goytain A, Ng T. “NanoString nCounter Technology: High-Throughput RNA Validation.,” In: Li H, Elfman J, editors. *Chimeric RNA: Methods and Protocols*. Methods in Molecular Biology. New York, NY: Springer US (2020). p. 125–139 doi: 10.1007/978-1-4939-9904-0_10

14. Bertrand G, Duprat E, Lefranc M-P, Marti J, Coste J. Characterization of human FCGR3B*02 (HNA-1b, NA2) cDNAs and IMGT standardized description of FCGR3B alleles. *Tissue Antigens* (2004) 64:119–131. doi: 10.1111/j.1399-0039.2004.00259.x
